# Mutual mate guarding with limited sexual conflict in a sex-role-reversed shorebird

**DOI:** 10.1093/beheco/arad084

**Published:** 2023-12-01

**Authors:** Johannes Krietsch, Mihai Valcu, Margherita Cragnolini, Wolfgang Forstmeier, Bart Kempenaers

**Affiliations:** Department of Ornithology, Max Planck Institute for Biological Intelligence, Eberhard Gwinner Str., 82319 Seewiesen, Germany; Department of Ornithology, Max Planck Institute for Biological Intelligence, Eberhard Gwinner Str., 82319 Seewiesen, Germany; Department of Ornithology, Max Planck Institute for Biological Intelligence, Eberhard Gwinner Str., 82319 Seewiesen, Germany; Department of Ornithology, Max Planck Institute for Biological Intelligence, Eberhard Gwinner Str., 82319 Seewiesen, Germany; Department of Ornithology, Max Planck Institute for Biological Intelligence, Eberhard Gwinner Str., 82319 Seewiesen, Germany

**Keywords:** extrapair paternity, mate guarding, *Phalaropus fulicarius*, red phalarope, sexual selection, short-term pair bond, social polyandry, sex-role reversal

## Abstract

Mate guarding is typically considered a male strategy to protect paternity. However, under some circumstances, females might also benefit from guarding their mate. Female mate guarding might be particularly important in socially polyandrous species in which females compete for access to care-giving males. Because males also benefit from being near their partner to avoid paternity loss, pair members may have a mutual interest in mate guarding in polyandrous species. We studied the time spent together and movements that lead to separation, as behavioral measures of mate guarding, in the classically polyandrous red phalarope (*Phalaropus fulicarius*). We equipped 64 breeding pairs with miniaturized telemetry loggers with GPS to assess variation in mate-guarding intensity in relation to breeding phenology and season, nest attendance, and the occurrence of extrapair paternity. We show that red phalarope pairs were almost continuously together in the days before clutch initiation with no sex bias in separation movements, indicating mutual contribution to mate guarding. Our results suggest that in red phalaropes, both pair members guard their mate, with limited sexual conflict arising through biases in the operational sex ratio and a trade-off with male nest attendance. We found no clear relationship between mate-guarding intensity and the occurrence of extrapair paternity. In this non-territorial socially polyandrous species, mutual mate guarding might be the process underlying the evolution of a brief but strong social pair bond, with no other purpose than producing a clutch for a care-giving male.

## Teaser

In a few species, referred to as “sex-role reversed,” females compete for males who provide most or all parental care. Using continuous individual tracking data of red phalaropes, we show that these shorebird pairs spent most of their time in close proximity until egg laying. Males may benefit from “mate guarding” to avoid paternity loss, while females benefit from avoiding mate takeover, which may have led to the evolution of a brief but strong social pair bond.

## INTRODUCTION

Mate guarding, behaviors that aim at preventing a mate from reproducing with others, has mainly been considered as a manifestation of sexual conflict (Parker 1974; Gowaty 1996; Zuk 2011; Arnqvist and Rowe 2013). In socially monogamous species, males risk losing paternity if their female engages in extrapair copulations, which causes selection favoring paternity assurance behavior in males. Male mate guarding is typically characterized by maintaining close proximity to the mate to prevent extrapair copulations leading to paternity loss and can be accompanied with or even replaced by frequent within-pair copulation (Møller and Birkhead 1991; Birkhead and Møller 1992; Harts et al. 2016). Females may incur a cost of mate guarding (e.g., reduced foraging efficiency; Davies 1985), and hence might benefit from escaping it. Alternatively, females may benefit from being guarded by their mate, for example, if it leads to a reduced risk of harassment or forced copulation by other males, of contracting a sexually transmitted disease, or of predation (Sheldon 1993; Kempenaers et al. 1995; Davis 2002; Low 2005; Rodríguez-Muñoz et al. 2011).

However, there are also circumstances in which selection can favor females to guard their social mate. For example, in socially facultatively polygynous systems with biparental care, females pay a cost when their mate obtains a secondary female if the male divides his effort between multiple nests (Slagsvold and Lifjeld 1994; Huk and Winkel 2006; Schlicht and Kempenaers 2021). The primary (first-mated) female thus benefits from guarding her mate to avoid or delay the settlement of a secondary female, if this ensures the male’s exclusive help with brood care (Kempenaers et al. 1995). Furthermore, mate guarding can be beneficial for both pair members, for example, if it reduces the risk of divorce, which can be costly for both sexes in socially monogamous species (Choudhury 1995). Mutual benefits of mate guarding have also been suggested as an explanation for the evolution of duets in song birds, although this is only one of several alternative hypotheses (Grafe and Bitz 2004; Hall 2009; Diniz et al. 2020).

Mutual mate guarding can also be expected in socially polyandrous species, in which patterns of mate guarding have rarely been studied. In this unusual mating system (described for < 1% of all species, Oring 1986; Owens 2002; Cockburn 2006), the typical sex roles are reversed, with males providing all parental care. The reproductive rate of females is limited by access to males, leading to competition among females for care-giving males. Thus, females benefit from guarding one (or multiple) males to avoid a takeover by another female. Because females mate with multiple males simultaneously or in close succession, males of socially polyandrous species face a higher risk of paternity loss compared to closely related socially monogamous species (Emlen et al. 1998; Schamel, Tracy, Lank, Westneat 2004; Safari and Goymann 2018; Krietsch et al. 2022). Thus, males may benefit from guarding their fertile female to avoid paternity loss, while females may also benefit from staying close to their mate if low confidence in paternity would lead to male desertion of the female’s clutch. In sum, mate guarding may be mutually beneficial.

Despite these mutual benefits of mate guarding in socially polyandrous species, sexual conflict can arise if trade-offs between mate guarding and other behaviors cause different optima for males and females (Arnqvist and Rowe 2013). Disentangling the selective forces on male and female mate-guarding behavior is therefore challenging, and both the social and environmental context has to be considered (Haneke-Reinders et al. 2020). Several factors can cause variation in mate-guarding intensity in males and females of socially polyandrous species. First, mate guarding is no longer beneficial for a female once her mate is committed to the clutch because it hinders searching for and mating with another male (i.e., a care giver for a subsequent clutch; Krietsch et al. 2022). Depending on the timing relative to the end of egg laying, this can cause a conflict with the female’s current mate, because it is in the male’s reproductive interest to keep mate guarding until the end of the female’s fertile period (i.e., the day on which the penultimate egg is laid; Birkhead and Møller 1992). This conflict might become stronger as the breeding season progresses because the number of males tending a clutch increases, which creates a stronger female-biased operational sex ratio and more intense female–female competition for mates (Tracy and Schamel 1988). Second, male mate guarding may trade-off with nest construction, early incubation, or nest protection (Safari and Goymann 2018). Third, for both sexes, mate guarding might trade-off with the pursuit of extrapair copulations (Hasselquist and Bensch 1991; Dickinson 1997; Wilson and Swaddle 2013). Males may adjust their investment in mate guarding depending on opportunities to sire extrapair young and on the risk of losing paternity in their own nest (Johnsen and Lifjeld 1995; Kokko and Morrell 2005; Wilson and Swaddle 2013). Similarly, females may try to evade mate guarding if they benefit from obtaining extrapair copulations (which remains unclear: Forstmeier et al. 2014; Brouwer and Griffith 2019). Fourth, in facultatively polyandrous systems, mate guarding might trade-off with investment into subsequent mates, as previously described in facultatively polygynous mating system (Hasselquist and Bensch 1991). For example, females might pursue extrapair copulations to improve the chances to quickly acquire a male for a subsequent clutch (Krietsch et al. 2022).

We studied variation in the intensity of mate-guarding behavior in the sex-role-reversed red phalarope (*Phalaropus fulicarius*). In this socially polyandrous species, females lay up to three clutches for different males during a short arctic breeding season, although in our study site, most females only laid one clutch (Schamel and Tracy 1977; Krietsch et al. 2022). Based on location data from 64 red phalarope breeding pairs, we describe two behavioral measures related to mate guarding: (1) the proportion of time the pair spent in close proximity, and (2) the number of movements initiated by the male or the female that clearly separate the pair members, as well as the distance of these flights (Parker 1974; Gowaty 1996).

The first aim of our study was to assess whether mate guarding is mainly driven by males (as a paternity protection behavior), by females (to retain a care giver), or by both (mutually beneficial). If mate guarding is mainly male driven, pairs should be in close proximity, and males should not (or rarely) initiate movements resulting in separation of the pair during the female’s fertile period. If mate guarding is mostly female driven, pairs should stay in close proximity immediately after pair formation, and females should not initiate movements that result in separation from the mate until the male is committed to tend the clutch, which does not necessarily coincide with the end of the female’s fertile period. Mate guarding could therefore be mutually beneficial for both sexes, until the fertile female starts pursuing other mating opportunities (Krietsch et al. 2022).

The second aim of our study was to investigate seasonal changes in mate-guarding intensity and to test whether polyandrous females or early-breeding females associated less with their mate during egg laying. If mate guarding is female driven and related to the risk of losing the care-giving male, we expect that mate guarding will be less intense early in the season (when most males are available) and become stronger as males start incubating, that is, as the operational sex ratio becomes more female biased (Tracy and Schamel 1988; Cosgrove et al. 2020). During the short Arctic breeding season, only early-breeding females have a chance to become polyandrous (Krietsch et al. 2022). Thus, early in the season, selection might favor females to quickly move on to attract a new partner, while later in the season, when the opportunity to find a new mate is low, females might benefit from associating longer with their current mate, to potentially quickly lay a replacement clutch, should their first clutch fail.

The third aim of our study was to assess seasonal changes in mate-guarding intensity in relation to time spent at the nest by the male. In red phalaropes, the nest is a simple scrape, but males add some nest material (small leaves) and create some cover by bending grasses over the nest (Mayfield 1979). Moreover, males might need to guard (cover) the nest once the first egg appeared to avoid egg predation (Smith et al. 2012). In theory, males might also need to guard against brood parasites, but there is no evidence for inter- or intraspecific brood parasitism in red phalaropes (Dale et al. 1999; Krietsch et al. 2022).

The fourth aim of our study was to investigate whether mate-guarding intensity during the fertile period differed between males that lost paternity and those that did not. Males that lost paternity might have guarded less intensely (i.e., extrapair paternity is a consequence of a lack of mate guarding), or they might have guarded more intensely if there was an indication that their mate was seeking extrapair copulations, making the best-of-a-bad-job (Kempenaers et al. 1995; Kokko and Morrell 2005).

## METHODS

### Study species and study site

We studied red phalaropes (*Phalaropus fulicarius*) in a 2.5 km^2^ plot of open wet tundra habitat near Utqiaġvik (formerly Barrow), Alaska (71°19ʹN 156°39ʹW) between late May and late July 2018 and 2019. Red phalaropes are non-territorial socially polyandrous birds that breed circumpolar in the Arctic (Tracy et al. 2020). In this species, the “typical” sex roles are reversed: females compete more strongly for mating opportunities and are larger and more brightly colored than males, and males provide all parental care. Females usually leave their mate directly after finishing their clutch and—if unpaired males are available—remate and lay another clutch for a subsequent male (sequential polyandry), with typically two, rarely three clutches produced per season (Schamel and Tracy 1977; Krietsch et al. 2022). Red phalaropes form short-term pair bonds that are characterized by close proximity between female and male, frequent within-pair copulations, and frequent communication with contact calls (Kistchinski 1975; Schamel and Tracy 1977). Both pair members aggressively defend their partner against approaching conspecifics, involving fights with both same- and opposite-sex individuals (Tracy et al. 2020). An earlier study showed that 33% of clutches (6/18) contained at least one extrapair offspring (Dale et al. 1999), but a recent study with a larger sample size found a substantially lower rate (11% of clutches, 37/334) with yearly variation between 0% and 19% (*n* = 8 years; Krietsch et al. 2022). Previous studies also suggested that the frequency of sequential polyandry was high (proportion of females that laid clutches for > 1 male: 44% [4/9 females]; Schamel and Tracy 1977; and 50% [3/6 females]; Whitfield 1995). However, a recent, more intensive study found a much lower rate, with, on average, 7% of females (11/162, range: 3–9% over 3 years) laying clutches for multiple males (Krietsch et al. 2022).

### Field procedures

We caught red phalaropes with handheld mist nests as soon as they arrived at the study site (total number of individuals: 203 in 2018 and 319 in 2019). Each captured individual was sexed based on plumage characteristics (Tracy et al. 2020), and banded with a U.S. Geological Survey metal band and a unique combination of four color bands that allowed us to identify and document the behavior of individual birds. The assigned sex of each individual was later confirmed with molecular methods (Krietsch et al. 2022).

After pairs were observed, we searched for nests daily across the study site with a team of 2–10 people by (1) following males or pairs that made scrapes or sat in scrapes, behavior typically shown a few days before the first egg was laid, (2) following females until they went to a scrape or nest to lay an egg, (3) following males after flushing them off a nest (accidentally or by rope dragging) or during a natural incubation break until they returned to their nest, and (4) by inspecting potential nest locations based on the GPS data of males (see below). Then, to avoid loss of data through predation, we collected all eggs and replaced them with dummy eggs that resembled real eggs in size, weight, and color. The collected eggs were hatched in an incubator, and the young brought back to an incubating male after blood sampling. This allowed us to determine the parentage (based on 30 microsatellite markers) of almost all eggs laid within the study site and of some within the surrounding area (for details, see Krietsch et al. 2022).

We determined clutch initiation dates (i.e., the date the first egg was laid) either by (1) subtracting one day for each egg in the nest for clutches found during egg-laying (assuming one egg per day was laid, 41% of nests), (2) subtracting one day for each egg in the clutch plus the mean incubation period of 17 days (0.8 SD; range: 15–20 days) for eggs hatching in the incubator (based on 58 nests with known laying date) or 19 days for naturally incubated clutches (Weiser et al. 2018) (34% of nests), or (3) subtracting the estimated developmental age based on flotation (Liebezeit et al. 2007) and clutch size for clutches that did not hatch (12% of nests), (4) by correcting dates estimated with method (2 or 3) with conflicting yet more reliable field observations (the number of eggs in the clutch; 10% of nests), or (5) based on nest visits by tagged males (3% of nests). For females known to lay multiple clutches (based on parentage analysis), we categorized each clutch as first, second, or third based on clutch initiation date.

All procedures were approved by the US Geological Survey Bird Banding Laboratory (permit number 23520), the Alaska Department of Fish and Game (permit numbers 17–149, 18–146, and 19-143), the US Fish and Wildlife Service (permit number MB210494-0), and the North Slope Borough and Ukpeaġvik Iñupiat Corporation.

### Tracking methods and data processing

We attached 3.5 g NanoRadioTag-3 (Milsar, Inc.) telemetry loggers with a solar panel on the back of some of the caught birds within the study area (2018: *N* = 101 of 210 caught in total, 53 males and 48 females, 2019: *N* = 202 of 323 caught individuals, 105 males and 97 females). Four males were equipped with a tag in both years, and three individuals received a second tag after the first one fell off. We focused on early-arriving birds and attached the majority of tags before the peak of clutch initiation (median attachment date: 14 June 2018 (range: 11–22 June) and 10 June 2019 (range: 5–27 June); median clutch initiation date: 23 June 2018 (*N* = 37 clutches) and 14 June 2019 (*N* = 100)). In 2018, we glued the tag, mounted on a piece of goatskin, on the back of the bird after cutting some body feathers. We used a flexible, high-adherence, nontoxic glue (Pattex Repair Extreme). In 12 cases, we either found the tag on the tundra or observed the bird without a tag after an average 5 days of attachment (range: 1–11 days). Therefore, in 2019, we used a backpack harness made out of medical-grade silicon tubing (DKA-795, Reichelt Chemietechnik GmbH). To ensure that the harness would eventually fall off, we connected the tag with the harness material using a single string of dissolvable sewing material (Novosyn Quick) as a weak link. No bird lost its tag during the study period in 2019.

Each tag was set to provide a GPS position every 10 min, with data stored on the tag until they were transmitted to a base station. We downloaded data daily for individuals who stayed within the study area, using a handheld base station with an omnidirectional antenna (~500 m range) or with a fixed directional antenna (~1500 m range) on a high pole. To maximize downloading data from individuals who had left the study area, we drove along the roads around Utqiaġvik once a day with a directional antenna mounted to a van. In 2019, we additionally downloaded data by flying a drone (Dji Mavic 2 Pro) with an attached omnidirectional antenna (~1000 m range) in or near the study area.

Overall, we retrieved data from 296 out of 303 tagged individuals (100 in 2018 and 196 in 2019). The seven remaining tags (one in 2018 and six in 2019) were never downloaded, most likely because these individuals had left the study area soon after release. In 2018, we obtained locations for up to 27 days (median = 4), and in 2019, for up to 51 days (median = 11). The difference between the years was due to the change in attachment method and to the shorter breeding season in 2018 (Krietsch et al. 2022). In 2018, we found 13 nests with a tagged male and 13 nests with a tagged female, with both individuals tagged for 11 nests. In 2019, we found 76 nests with a tagged male and 66 nests with a tagged female, with both individuals tagged at 56 nests (four of these nests were social pairs renesting after the failure of the first clutch).

To assess variation in mate-guarding intensity, we used location data from 64 unique social pairs (63 unique males and 60 unique females) between the pre-laying and the end of the laying period (68 nests, 10 from 2018 and 58 from 2019). For the four pairs that produced two clutches (first and replacement clutch), we assigned all data until the failure of the first clutch to the first clutch and all data thereafter to the second clutch.

For each of the breeding individuals, we first selected all location data from the moment the individual had been released until the last data had been sent or until the individual had reached the position where the tag fell off. We then filtered out implausible positions, that is, (1) those > 500 km away from the capture site, 821 of 359,547 positions, 0.2%), (2) those that implied a faster speed than the maximum speed recorded in a continuous track (>105 km/h, 27 positions, <0.01%), and (3) single outliers (one location > 2.5 km away from previous and subsequent positions, which were within 100 m, excluding 23 positions, <0.01%). Thus, we used a filtered dataset of 358,676 positions.

### Data analysis

All data were analyzed with R version 4.2.2 (R Core Team 2023) using RStudio (RStudio Team 2023). We fitted generalized linear mixed models (GLMMs) using the package “glmmTMB” (Brooks et al. 2017) with a binomial distribution and logit link when the dependent variable was binary, with a beta-binomial distribution and logit link when the dependent variable was a proportion and with a Gaussian distribution when the dependent variable was continuous. In all models, we included nest ID as a random effect, allowing for random slopes over the day relative to clutch initiation. We assessed model residual diagnostics using the package “DHARMa” (Hartig 2022). We extracted effect sizes using the package “effects” (Fox and Weisberg 2018). We present back-transformed mean effect sizes with standard error (SE) in the text and full model summaries in the Supplementary Material. All figures were created using the package “ggplot2” (Wickham 2016).

#### Time spent together

We used the filtered location data to determine when pair members or any two opposite-sex individuals were together, as follows. First, we calculated for each male-female pair the distances between locations that were recorded simultaneously, that is, within maximally 10 min of each other (in 95% of cases, this time gap was within 5 min of each other, median 2.6 min). Then, we used three rules to define whether two individuals were “together” at a given time. Two individuals were defined as being together if, first, the distance between their locations was smaller than a dynamic threshold of 30 m or more (the dynamic threshold allows for movements that happen during the time gap mentioned above; details described in Supplementary Figure S1), and, second, in a bout of successive locations “together” (below the dynamic threshold) at least one distance was smaller than 30 m (fixed threshold). If the last locations of the pair members in a bout of “together” observations were > 30 m apart, we defined the two individuals as “not together” in this last instance before separating. The latter was necessary, to distinguish flights that resulted in separation from flights that the birds did together (being together once the flight has come to an end; Supplementary Figure S1). Taken together, these three rules yielded a classification that corresponds with our intuitive judgment (see Supplementary Video). The proportion of time spent together on a given day was then calculated as the number of locations where the pair members were “together” divided by the total number of recorded locations for the pair on that day.

We used this “time spent together” variable as a proxy for mate-guarding intensity because it represents times when pair members were in close enough proximity to interfere when a conspecific would approach the mate. Note that mate guarding is not mutually exclusive with other behaviors, including foraging and those related to pair bonding (courtship and copulation).

To assess the effect of phenology and season on the time spent together, we fitted GLMMs with the proportion of time spent together per day as the dependent variable and day relative to clutch initiation (i.e., the day on which the first egg was laid = 0) and clutch initiation date (the actual date, standardized by subtracting the mean of each year) as explanatory variables. In all models, day relative to clutch initiation and clutch initiation date were fit first as covariates with a quadratic effect. When the quadratic effect was nonsignificant, we only included the linear effect in the final model. Because the birds’ behavior may change after the start of egg laying, we fitted separate models for the period before clutch initiation (day −5 to −1) and during egg laying (day 0–3, most females laid four eggs). We initially tested for daily variation by fitting both models with the binary variable “together” (yes/no at a given 10-min interval) as the dependent variable and time (as sinus and cosinus) as additional explanatory variable. Daily variation in the time spent together was biologically irrelevant in the period before clutch initiation (daily maximum minus daily minimum = 1%; Supplementary Table S1), but more pronounced during egg laying (max-min = 10%; Supplementary Table S2), with birds spending more time together midday (71%; ± 3.6 SE) compared to midnight (61%; ± 4.1 SE). Note that during the study period, there is 24-h day light and birds are active around the clock (Steiger et al. 2013). Because we were interested in seasonal variation and because the models using data at 10-min intervals suffer from temporal autocorrelation, we used the proportion of time spent together per day in subsequent models. We also fit year as an additional explanatory variable, but we excluded it in the final model because there were no significant differences in the proportion of time spent together between the years for both periods (day −5 to −1: *P* = 0.30; day 0–3: *P* = 0.83; Supplementary Tables S3 and S4; Supplementary Figure S3).

We compared the proportion of time spent together by breeding pairs and by non-breeding “random” pairs (two opposite-sex individuals that did not breed together), using the same two models, but including pair type (breeding pair or random pair) in interaction with day relative to clutch initiation and clutch initiation date as explanatory variable. We fitted an additional model for the period after the egg-laying period (days 4–10) with the same structure. For the random pairs, we only included pairs of opposite-sex individuals if (1) we had location data for at least 50% of a given day and (2) the pair had at least one 10 min period “together.” The latter excludes pairs of individuals that were never in each other’s neighborhood (e.g., using the same foraging site). To obtain comparable data given variation relative to clutch initiation of the breeding pairs, we randomly sampled 50 non-breeding pairs for each day relative to the clutch initiation date of each focal female.

#### Movements away from the mate

We defined the individual that was responsible for separating the pair as the individual that moved the furthest between two 10-min periods with a change from “together” to “not together,” as defined above. Note that this criterion may not always identify the correct individual that left the partner, because individuals can move undetected within the 10-min interval. However, in 72% of the cases when a pair split, one individual stayed stationary (i.e., within 30 m), and in the other 28% of the cases, the individual that was assigned as responsible for the separation moved on average 64 m (median) further than the other pair member.

We used all separation events and fitted GLMMs with the binary variable “female move” (yes/no, whereby “no” means that the male was responsible for the separating move) as the dependent variable and with day relative to clutch initiation and clutch initiation date as explanatory variables. Again, we fitted separate models for the period before clutch initiation (days −5 to −1) and during egg laying (day 0–3). Additionally, we fitted a GLMM with the distance moved away when separated as the dependent variable and with the same explanatory variables but also including the sex of the moving bird.

#### Time spent at the nest with and without the mate

We defined an individual as “at the nest” if either (1) its position was within 15 m of the known nest coordinates or (2) when the individual was “together” with the partner, and the partner was within 15 m of the nest. For each day, we then calculated the proportion of positions an individual was at the nest, with or without its mate, and overall. We also determined the proportion of the day the focal individual was not together with its mate and not at the nest.

For males and females separately, we fitted GLMMs with the proportion of time spent at the nest per day during the period of egg laying (day 0–3) as the dependent variable and day relative to clutch initiation and clutch initiation date as explanatory variables.

#### Mate-guarding behavior in relation to extrapair paternity and social polyandry

We evaluated whether social pairs with extrapair sired eggs in their clutch differed in mate-guarding behavior (time spent together and proportion of separating moves by the male or the female) from pairs without extrapair paternity. We fitted GLMMs with the proportion of time spent together per day or “female move” (yes/no, whereby “no” indicates that the male was responsible for the separation) as the dependent variable and extrapair paternity (yes/no) and day relative to clutch initiation and clutch initiation date as explanatory variables. We fitted separate models for the potential fertile period of the female: before clutch initiation (day −5 to −1) and during clutch initiation (day 0–2, excluding the last day of laying).

As our sample size of polyandrous females was small, we only visually compared the time the female spent together with her first and second mate relative to the initiation of the two clutches.

## RESULTS

### Time spent together in relation to clutch initiation and season

**Figure 1:**
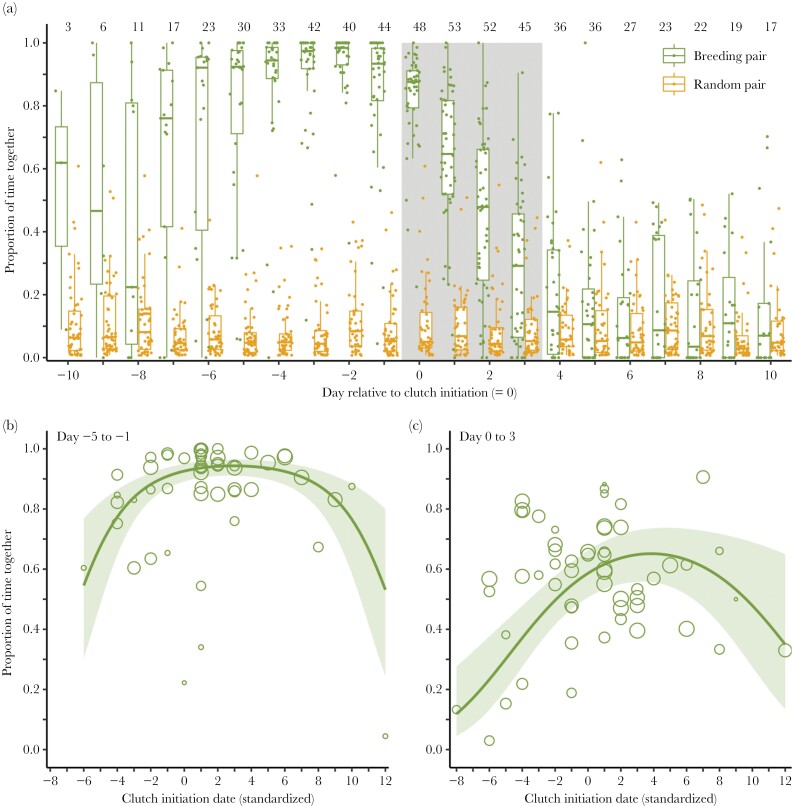
(a) Proportion of time red phalarope breeding pair members spent together in relation to day relative to the initiation of the clutch of the focal pair, and compared to random (non-breeding) pairs of opposite sex individuals. The data are from a total of 64 breeding pairs (68 clutches, from 63 males and 60 females) and 50 random pairs (see methods). Shown are box plots with the median (center line), 25–75th percentile (limits), minimum and maximum values without outliers (whiskers), and raw data for each day by breeding pair (dots). The gray-shaded area indicates the egg-laying period, assuming a typical clutch of four eggs. Numbers on top indicate the number of pairs with data for each day. (b, c) Proportion of time red phalarope breeding pair members spent together in relation to their date of clutch initiation, standardized by subtracting the mean of each year, during the pre-laying period (b) and the laying period (c). Model estimates (lines), 95% confidence intervals (shaded areas) and the mean proportion by pair (dots) show that early and late-breeding pairs spent less time together than those breeding in the middle of the season. Dot size reflects the number of pairwise observations for each breeding pair (range: 14–686 observations). See Supplementary Tables S5 and S6 for model descriptions.

We caught and observed most red phalaropes in pairs, suggesting that individuals were already paired before or soon after they arrived at the study site. The tag data show that members of a future breeding pair were often together almost immediately after tag deployment and release (Supplementary Figure S4). Before clutch initiation, breeding pairs typically spent most of their time together (percentage of time together from day −5 to −1: 90% ± 2.2 SE; Supplementary Table S5 and Figure 1a) and moved together between potential foraging or nesting sites (see Supplementary Videos of three pairs as examples). The proportion of time spent together peaked 2 days before clutch initiation with 34 out of 40 breeding pairs for which we had data on this day spending more than 90% of the time together (94% ± 1.4 SE; Figure 1a). When egg laying started the time spent together was still high (day 0: 84% ± 2.1 SE), but it decreased rapidly throughout egg laying (day 3: 28% ± 4.9 SE; Supplementary Table S6). Throughout the pre-laying period, as well as during egg laying, breeding pairs spent much more time together than random pairs of opposite-sex individuals that were at least once together but did not breed together (breeding pairs day −5 to −1: 94% ± 0.7 SE; randomized pairs: 12% ± 1.4 SE, *P* < 0.001, Supplementary Table S7; breeding pairs day 0–3: 58% ± 3.2 SE; randomized pairs: 11% ± 1.5 SE, *P* < 0.001; Figure 1a, Supplementary Table S8). In the days after clutch completion, breeding pairs spent little time together, and as little as random pairs (breeding pairs day 4–10: 10% ± 1.1 SE; randomized pairs: 11% ± 1.2 SE, *P* = 0.13, Supplementary Table S9; Figure 1a).

The proportion of time breeding pairs spent together was not only changing with the day relative to clutch initiation but was also influenced by the season (i.e., the actual date at which the clutch was initiated). In the pre-laying period (days −5 to −1), breeding pairs spent the most time together (>90%) in clutches that were laid in the middle of the season (between one day before and 7 days after the mean clutch initiation date of a given year; Figure 1b). Breeding pairs that laid earlier or later than this period spent less time together (6 to 2 days before the mean clutch initiation date: 74% ± 6.6 SE; 8–12 days after the mean: 73% ± 8.6 SE; Supplementary Table S5, Figure 1b). A similar pattern was found in the laying period (days 0–3), when breeding pairs that laid their first egg 1 day after the mean clutch initiation date spent the most time together (Supplementary Table S6, Figure 1c).

### Movements away from the mate in relation to clutch initiation and season

**Figure 2:**
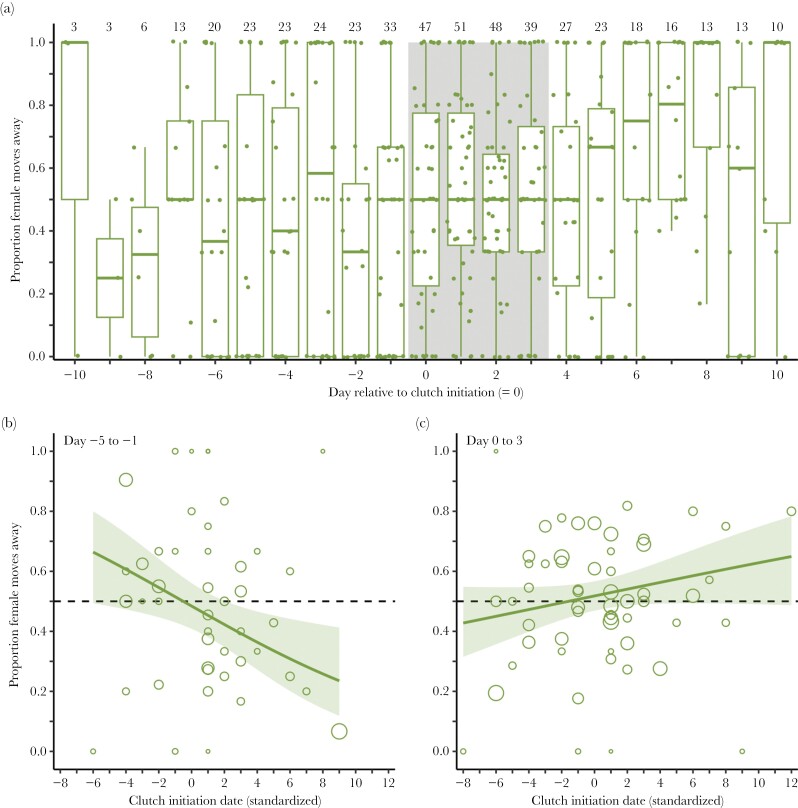
(a) Proportion of the total number of separating flights in which the female moved away from the male in relation to the start of egg laying, that is, day relative to the initiation of the clutch of the focal pair. Shown are box plots with the median (center line), 25–75th percentile (limits), minimum and maximum values without outliers (whiskers), and raw data for each day by breeding pair (dots). The gray-shaded area indicates the egg-laying period, assuming a typical clutch of four eggs. Numbers on top indicate the number of pairs with data for each day. (b, c) Proportion of the total number of separating flights in which the female moved away from the male in relation to the clutch initiation date (standardized by subtracting the mean of each year), during the pre-laying period (b) and the laying period (c). Shown are model estimates (lines), 95% confidence intervals (shaded areas) and the mean proportion by pair (dots). Dot size reflects the number of pairwise observations for each breeding pair (range: 1–37 observations). See Supplementary Tables S10 and S11 for model descriptions.

Given that pairs spent most of the time together before clutch initiation, they only separated rarely in this period (day −5 to −1; mean by pair and day: 1.9 times, range: 0–9 times, Supplementary Figure S5). Pair separation became more frequent during egg laying (day 0–3; mean by pair and day: 4.6 times, range: 0–17 times). Males and females were more or less equally responsible for movements that resulted in the separation of the pair both before and during laying (proportion of separating moves made by the female, day −5 to −1: 45% ± 5.0 SE; day 0–3: 52% ± 3.3 SE; Supplementary Tables S10 and S11, Figure 2a).

However, separation movements varied across the season, at least during the pre-laying period. In pairs that initiated laying early in the season, separating movements were mostly initiated by females (59% ± 6.2 SE, pairs with clutch initiation before the mean), while for later pairs, separations were mostly initiated by males (35% ± 5.5 SE female initiated, pairs with clutch initiation on or after the mean laying date, *P* = 0.01; Supplementary Table S10, Figure 2b). The effect of the clutch initiation date was less clear and not statistically significant for movements during the laying period (47% ± 4.3 SE vs. 58% ± 4.8 SE, *P* = 0.19; Supplementary Table S11, Figure 2c).

During both the pre-laying and the laying period, the distance of the movement that led to the partners being separated was independent of the sex of the individual responsible for the movement and only slightly larger during laying (days −5 to −1: males: 140 m ± 15.2 SE, females:170 m ± 15.8 SE, *P* = 0.07; Supplementary Table S12; days 0–3: males: 194 m ± 25.8 SE, females: 216 m ± 25.8 SE, *P* = 0.16; Supplementary Table S13, Supplementary Figure S8).

### Time spent at the nest with and without the mate

**Figure 3:**
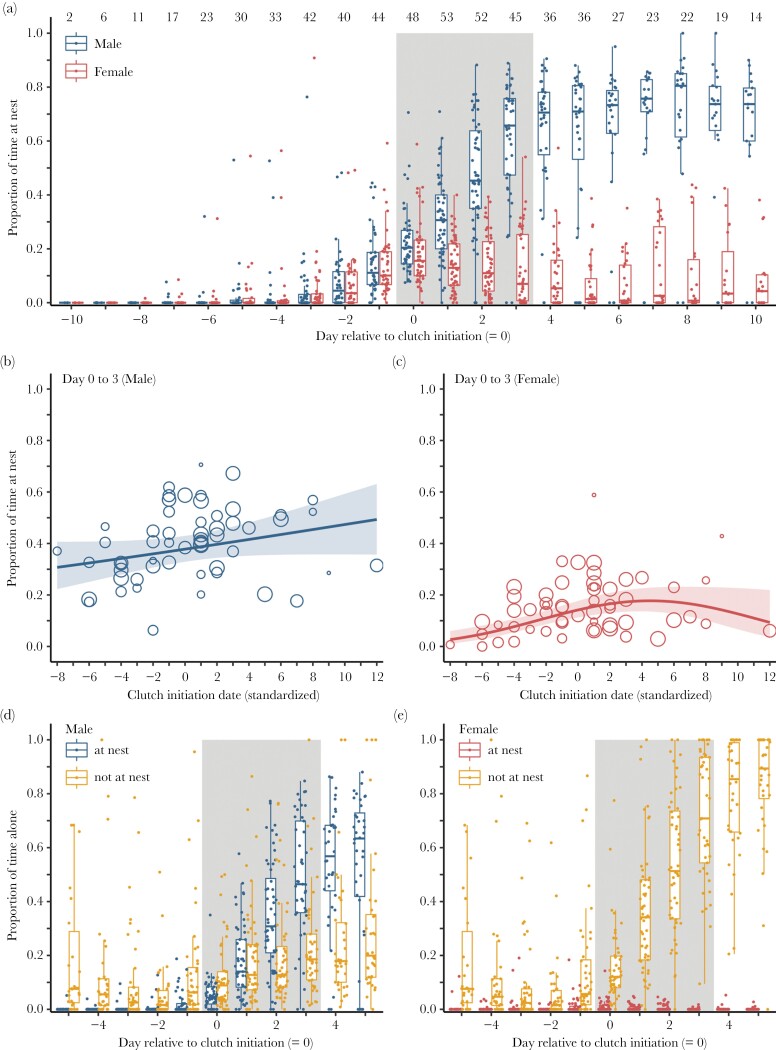
(a) Proportion of time male and female red phalaropes spent at the nest (location within 15 m or together with mate who is within 15 m from the nest) in relation to the start of egg laying, that is, day relative to the initiation of the clutch of the focal pair. Shown are box plots with the median (center line), 25–75th percentile (limits), minimum and maximum values without outliers (whiskers), and raw data for each day by sex for each breeding pair (dots). The gray-shaded area indicates the egg-laying period, assuming a typical clutch of four eggs. Numbers on top indicate the number of pairs with data for each day. (b, c) Proportion of time male (b) and female (c) red phalaropes spend at the nest in relation to the clutch initiation date (standardized by subtracting the mean of each year). Shown are model estimates (lines), 95% confidence intervals (shaded areas) and the mean proportion for each sex (dots). Dot size reflects the number of observations for each sex (range: 14–558 observations). See Supplementary Tables S14 and S15 for model descriptions. (d, e) Proportion of time male and female red phalaropes spend alone at the nest and away from the nest in relation to egg date. Shown as in (a).

Most red phalarope pairs only started spending time at their nest location three days before clutch initiation (mean: 2.6 days before clutch initiation, range: −7 to −1; *N* = 28 pairs with at least one day of data before the day of the first nest visit). On the day before clutch initiation, both sexes spent around 10% of their time at the nest (Figure 3a). On the day of clutch initiation, females spent the longest time at the nest overall (median: 16% of their total time), but this time decreases rapidly over the laying period to 7% on the last day of laying (Figure 3a). In contrast, males spent an increasing amount of time at the nest (from 20% on day 0 to 66% on day 3), suggesting a gradual start of incubation (Figure 3a).

How much time males and females spent at the nest was also influenced by the timing of clutch initiation within the season. Males whose female started laying relatively early in the season spent less time at the nest during the laying period (before or at the date of mean clutch initiation: 34% ± 3.5 SE; Supplementary Table S14, Figure 3b) compared to males whose female laid later in the season (after the mean clutch initiation date: 44% ± 4.4 SE). Females spent somewhat less time at the nest in early and late clutches, with a “peak” around the mean clutch initiation date (Supplementary Table S15, Figure 3c).

During the egg-laying period, males and females spent an increasing amount of time separated from their partner (Figure 1a). Males that were away from their partner spent most of this time at the nest (Figure 3d), suggesting a potential trade-off between nest attendance and mate guarding. Conversely, if females were alone, they were virtually always away from the nest (Figure 3e).

### Mate-guarding behavior in relation to extrapair paternity and social polyandry

**Figure 4:**
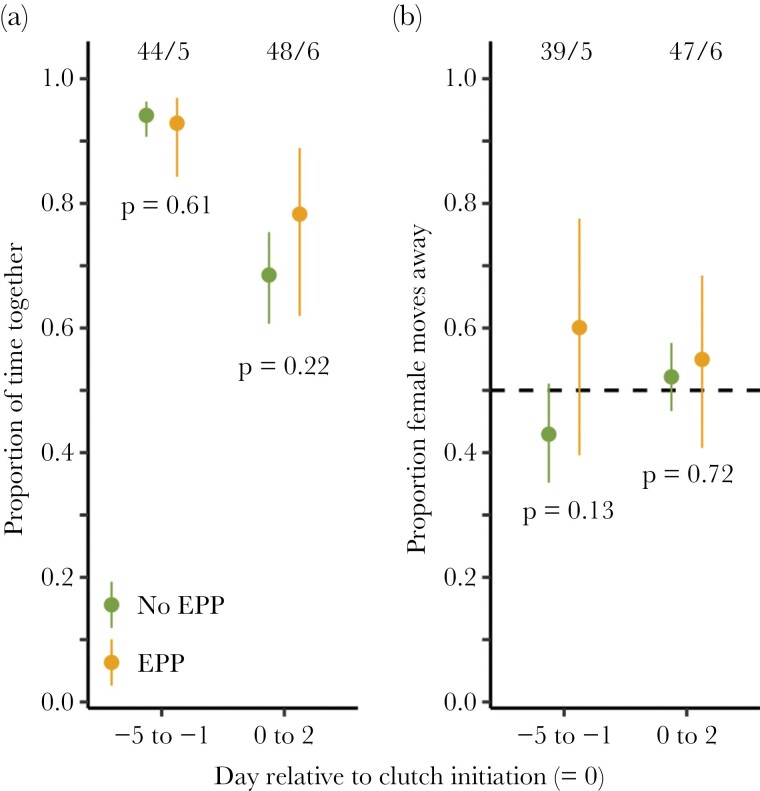
(a) Proportion of time red phalarope breeding pair members spent together during the fertile period of the female for pairs with and without extrapair paternity (EPP, defined as whether at least one egg in the clutch was sired by an extrapair male). (b) Proportion of the total number of separating flights in which the female moved away from the male for pairs with and without extrapair paternity. Shown are model estimates and 95% confidence intervals for the pre-laying period (days −5 to −1) and for the laying period during which the female was still fertile (days 0–2). Numbers on top indicate sample sizes (the number of pairs in each category). *P* values for each comparison are given below the estimates. See Supplementary Tables S16–S19 for model descriptions.

During the pre-laying period, breeding pairs with extrapair paternity (at least one extrapair sired egg in the clutch) spent a similar amount of time together compared to pairs without extrapair paternity (Figure 4a, Supplementary Table S16 and Supplementary Figure S7a). In the fertile period during egg laying, pairs with extrapair paternity spent somewhat more time together than those without, but the difference is not significant (*P* = 0.22, Figure 4a, Supplementary Table S17).

In pairs with extrapair paternity, females moved away from their mate more often than the other way around before clutch initiation, but this difference was not significant (*P* = 0.13, Figure 4b, Supplementary Table S18 and Supplementary Figure S7b). In the fertile period during egg laying, there were no differences between the sexes (Figure 4b, see also Supplementary Table S19).

**Figure 5:**
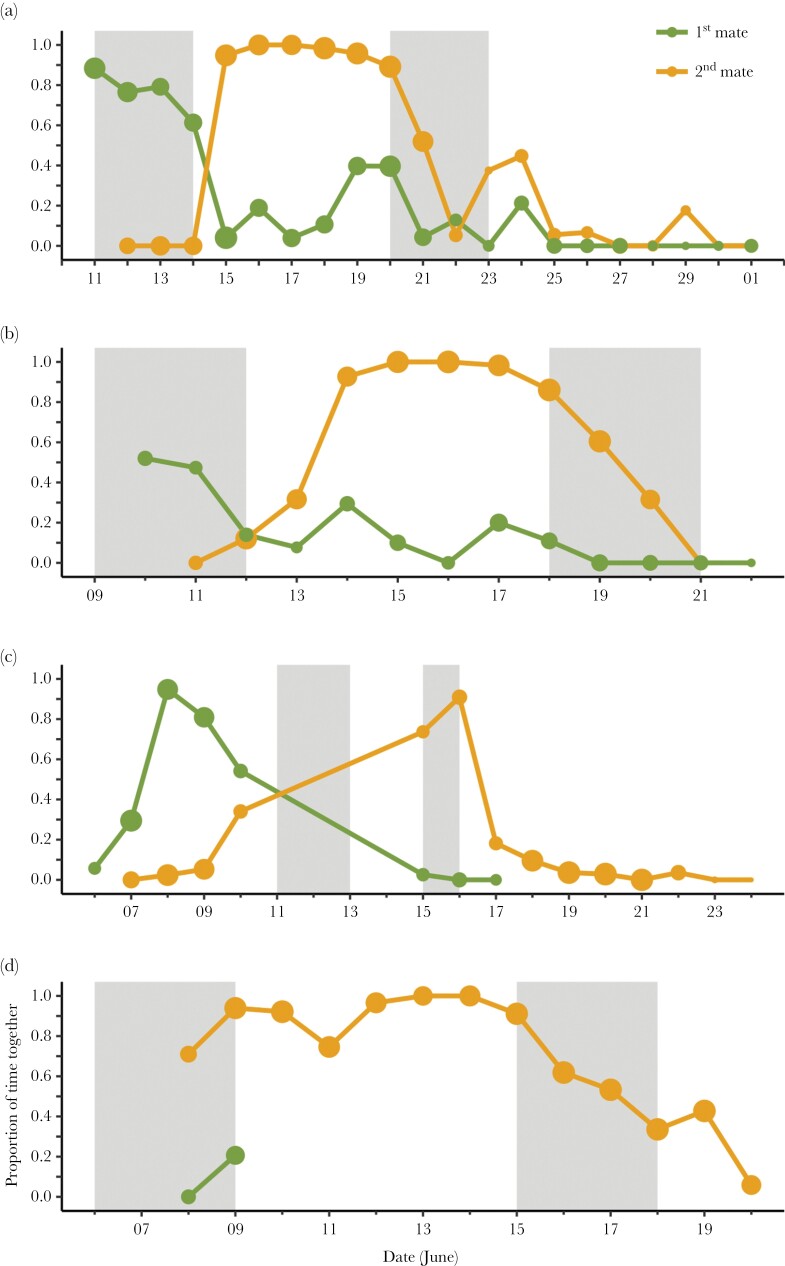
The proportion of time a polyandrous red phalarope female (*N* = 4, a–d) spent together with her first (green) and second (orange) social mate across the season. The gray-shaded areas indicate the egg-laying periods for the first and second clutch. Clutch size was four, except for the female in (c), who laid only three and two eggs, respectively. Dot size reflects the number of observations (10-min periods; range: 1–142).

We obtained location data from four polyandrous females and both of their social mates. One female spent more than 60% of her time together with the first mate before switching abruptly to the second mate after the completion of her first clutch (Figure 5a). The other three polyandrous females already spent some time together with the second mate while or even before egg laying for the first clutch (Figure 5b–d).

For three socially polyandrous females (Figure 5a–c), we had location data from her and her first mate during the laying of the second clutch. These data show that those females still spent some time in close proximity to their first social mate during this period, despite the fact that these males were incubating the first (and not the second) clutch.

## DISCUSSION

In this study of a socially polyandrous, sex-role-reversed species, we found multiple indications suggesting that both sexes guard their mate in a context-dependent manner, with females likely guarding males to ensure a care taker for their clutch and males guarding females to ensure their paternity. Mate guarding was most intense in the days just before the female laid the first egg and during the population-wide peak of clutch initiation when competition for mates was presumably the strongest (Figure 1). During the female’s laying period, the association between the male and the female rapidly dissolved, with males spending more time at the nest and females presumably searching for subsequent males (Figure 3). We found no clear link between mate-guarding intensity and the occurrence of extrapair paternity, but only few clutches contained extrapair sired eggs (Figure 4).

Our study suggests mutual mate guarding in red phalaropes particularly before clutch initiation. Red phalaropes spent almost all the time together with their mate, and on average, there was no sex bias in separation movements (Figures 1 and 2). While high-intensity mate guarding has also been described for many socially monogamous bird species (e.g., Birkhead 1979; Gowaty and Plissner 1987; Pinxten et al. 1987; Johnsen and Lifjeld 1995; Komdeur 2001; Hoi et al. 2011), the observed patterns are typically driven by the behavior of the male. In these species, the male typically follows his fertile female, and the majority of separation events are initiated by the female. Intense mate guarding has also been described in the closely related red-necked phalarope *Phalaropus lobatus*, which is also socially polyandrous with male-only care (Schamel, Tracy, Lank, Westneat 2004). In this study, behavioral observations showed that males moved more often toward the female than the other way around, but both sexes were equally likely to move away from their mate. However, Schamel, Tracy, Lank, Westneat (2004) only discussed mate guarding from the male perspective as a paternity protection behavior.

Several behavioral observations of social pairs (see Krietsch et al. 2022) provide additional support that in phalaropes both males and females actively guard their mate. (1) In 153 cases in which the sex that initiated the flight could clearly be identified, 58% (88/153) of flights were initiated by the female, and in 82% (72/88) of those cases, the male followed his female. Males initiated 42% (65/153) of the flights, and in 75% (49/65) of the cases, the female immediately followed her partner. (2) We recorded 144 aggressive interactions of pair members toward one or more other individuals. Aggressive interactions were initiated slightly more by females compared to males (males: 43%, 62/144; females: 57%, 82/144; both pair members: 6%, 9/144). Both male and female aggression was mainly directed toward another female (males: 66%, 40/61; females: 57%, 46/80), and less often against another male (males: 41%, 25/61; females: 42%, 34/80). The observation that the pair (i.e., both pair members) responded aggressively toward other individuals that approached them, which was also observed by Schamel and Tracy (1977) and also in red-necked phalaropes (Tracy and Schamel 1988), suggests that they signal unavailability to other potential partners. (4) As reported for red-necked phalaropes (Schamel, Tracy, Lank, Westneat 2004), red phalarope pairs copulated frequently, and copulations were initiated by both sexes. In 113 cases in which the initiating sex could be unequivocally identified, the male initiated the copulation with a “whirr-flight” in 82 cases (73%), while the female first lifted her tail and crouched in the other 31 cases. (5) Pair members use contact calls whenever direct visual contact is lost (Tracy et al. 2020). Frequent use of contact calls in phalaropes may reflect mutual mate guarding, as has been hypothesized for the evolution of duet songs in some birds (Grafe and Bitz 2004; Hall 2009; Diniz et al. 2020), although in territorial species, it is difficult to distinguish from mutual territory defense. Because red phalaropes are non-territorial, this alternative hypothesis can be excluded.

We found no clear relationship between mate-guarding intensity and the occurrence of extrapair paternity. Pairs with extrapair paternity spent a similar amount of time together before clutch initiation and slightly more time together during laying (nonsignificant), compared to pairs without extrapair paternity, but extrapair paternity was rare (Figure 4). In the period before clutch initiation, females that had extrapair sired eggs were responsible for somewhat more separation movements, but again the difference was not significant. During laying both pair members initiated an equal proportion of separation movements. Low rates of extrapair paternity in the population (11% of nests [37/334]; Krietsch et al. 2022) suggest that female red phalaropes do not typically try to escape male mate guarding. Previous observations also suggested that red phalarope females rarely interact with extrapair males, and they were never seen copulating outside the pair bond before clutch initiation (Krietsch et al. 2022).

Mate guarding was most intense during the peak clutch initiation period, when the conditions were presumably best for breeding and competition for mates likely the strongest. Early in the breeding season, pair members spent less time together before clutch initiation, and males were less likely to leave their female during this period (Figures 1 and 2), suggesting that mate guarding at this time could be more male driven. This pattern could be due to a male-biased sex ratio early in the season, with more intense male–male competition for females (Tracy and Schamel 1988). Cosgrove et al. (2020) reported a male-biased operational sex ratio early in the 2018 breeding season on a study site close to ours, which could reflect a male-biased adult sex ratio in the population and, therefore, generally high male availability (Schacht et al. 2022; Kappeler et al. 2023). With the progression of the season, each female that completes a (first) clutch will immediately rejoin the mating pool, whereas their male partner starts incubation and is therefore excluded from the mating pool. Therefore, over time, the operational sex-ratio will become more female-biased and female–female competition should become more intense (Lank et al. 1985; Tracy and Schamel 1988; Schamel, Tracy, Lank 2004). These changes in mate availability could explain why later in the breeding season, pairs spent less time together before clutch initiation and separation movements become more male biased (Figures 1 and 2), suggesting that mate guarding becomes more female driven. Males, as the only providers of parental care, may also have a decreasing interest in accepting a clutch, because of the lower prospect of chick survival later in the season (Saalfeld et al. 2021). Thus, seasonal effects on the operational sex ratio and on the likelihood of successful breeding can affect male and female investment in mate guarding.

After egg laying started, the close association between pair members rapidly dissolved (Figure 1). During this period, separation movements showed no sex bias, suggesting little sexual conflict (Figure 2). During the day of clutch initiation, most pairs were still together more than 80%, and both partners mainly visited the nest location together. Thereafter, males increasingly spent time alone at the nest, while females mostly foraged or interacted with a potential subsequent partner away from the nest (Figure 5). Male mate guarding, from a paternity assurance perspective, should be continued until extrapair copulations can no longer fertilize an egg, which should be up to the day on which the penultimate egg is laid (Birkhead and Møller 1992). However, mate-guarding intensity clearly decreased earlier, suggesting a potential trade-off for males between mate guarding and nest attendance. Nest attendance might become important because of nest-building activity or to reduce predation risk (i.e., by reducing exposure of the eggs), as has been shown for other species (Schleicher et al. 1993; Safari and Goymann 2018). Alternatively, the probability that copulations during the egg-laying period lead to fertilization of an egg in the clutch might already be low. While some sperm may reach the infundibulum soon after insemination, it typically takes longer, and the likelihood of fertilization is highest for copulations that occur 2–3 days before the egg is laid (Birkhead and Møller 1993). Given that red phalaropes typically lay four eggs, this could also explain why within-pair copulations become less frequent once laying starts (Krietsch et al. 2022).

From the female’s perspective, the benefits of mate guarding might also be lower after clutch initiation, if the probability of male desertion is low once egg laying has started. Females might then leave their social partner to increase the probability of finding a subsequent mate. Indeed, behavioral observations show that red phalarope females strongly increase the amount of extrapair interactions and extrapair copulations directly after clutch initiation, which can be interpreted as mate acquisition behavior and lead to a rapid subsequent reproductive event (Krietsch et al. 2022). This could also explain why pairs spent less time together after laying has started earlier in the season because during that period, females are more likely to find another mate and become socially polyandrous. Hence, females breeding earlier in the season might invest more in finding a second mate compared to females laying later in the season. After the population peak in clutch initiation, when female–female competition for a few remaining males is likely strongest, females might benefit from spending more time with the current mate, for example, to reduce male harassment by other females around the nest (Kistchinski 1975). In accordance with this hypothesis, both sexes spent more time at the nest later in the breeding season (Figure 3c,d).

In conclusion, in the socially polyandrous sex-role-reversed red phalarope, both pair members may benefit from mate guarding. Males would benefit from guarding their mate to protect their paternity, while females need to secure a male that cares for their clutch. To achieve this, females need to deter competitors (to avoid mate takeover), but they might also need to assure the male that he sired the eggs (to avoid male desertion). These male and female-specific reproductive interests would create the circumstances in which an exceptionally short and intense social pair bond with affiliative behavior and mutual mate guarding could have evolved. Under mutual mate guarding, the cost of mate guarding for either sex is presumably lower than if only one sex guards the other. These costs can be further reduced if the presence of two individuals improves foraging success (rather than hinders it) or leads to increased vigilance against predators. Mutual interest in staying close to the mate seems particularly strong during the peak breeding season, but the potential for sexual conflict might increase early and late in the breeding season. This seasonal effect is likely linked to shifts in the operational sex ratio, which creates a situation in which the less common sex becomes more valuable for the more common sex. Mate guarding has primarily been studied from the male perspective as a paternity-guarding mechanism, and disentangling the selective forces on male and female behavior in a “shared trait” is challenging. In this study, we show that to understand variation in mate-guarding behavior, it is important to consider both the male and female perspective, as well as context-dependent changes in reproductive interests of both males and females.

## Supplementary Material

arad084_suppl_Supplementary_Video

arad084_suppl_Supplementary_Material

arad084_suppl_Supplementary_Video_Caption

## Data Availability

Analyses and figures reported in this article can be reproduced using the data and code provided by Krietsch et al.(2023).
