# Supplementary material for: Mutual mate guarding with limited sexual conflict in a sex-role-reversed shorebird
Source: Behav Ecol. 2023 Dec 1;35(1):arad084. doi: 10.1093/beheco/arad084 (PMC10773304; doi:10.1093/beheco/arad084)
Supplement: arad084_suppl_Supplementary_Material [file arad084_suppl_supplementary_material.pdf]

Mutual mate guarding with limited sexual conflict in a sex-role reversed shorebird

Electronic supplementary material

Contents

Supplementary Figures 2
Figure S1. 3
Figure S2. 5
Figure S3. 6
Figure S4. 8
Figure S5. 9
Figure S6. 10
Figure S7. 11
Supplementary Tables 12
Table S1........ 12
Table S2........ 13
Table S3........ 14
Table S4........ 15
Table S5........ 16
Table S6........ 17
Table S7........ 18
Table S8........ 19
Table S9........ 20
Table S10........ 21
Table S11........ 22
Table S12........ 23
Table S13........ 24
Table S14........ 25
Table S15........ 26
Table S16........ 27
Table S17........ 28
Table S18........ 29
Table S19........ 30

Supplementary Figures

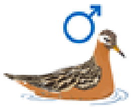

Interpretation of bouts of subsequent locations

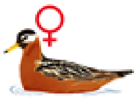

Each of the four scenarios below show three locations for the male (blue, A_1-A_3) and the female (red, B_1-B_3) of a pair, that were recorded in 10-min intervals, with a 3-min delay for the female locations. The red and blue lines illustrate the actual track of the two individuals. The light red or light blue area around each point indicate a 15 m radius (level of accuracy) and the black lines show the distance between the male and female locations at a given time interval (e.g. $d(A_1B_1)$). The dotted circles represent different sites where the birds spent a longer amount of time.

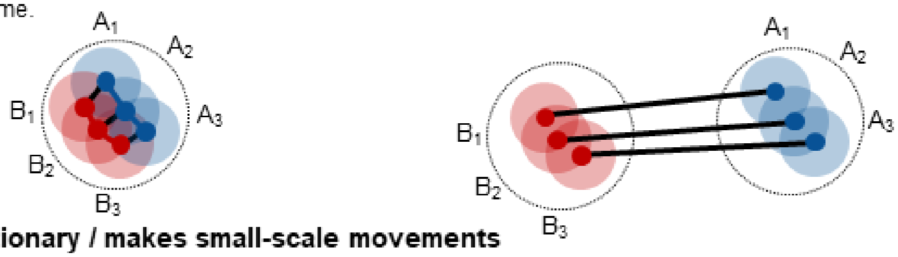

① **Pair is stationary / makes small-scale movements**

If males and females stay more or less stationary during the 3 subsequent 10-min intervals, a 30 m threshold would be sufficient to classify birds as "together" (left) or "not together" (right).

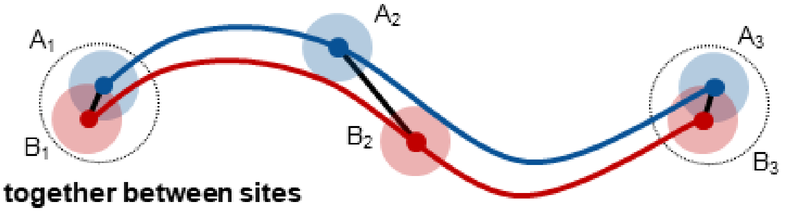

② **Pair moves together between sites**

A simple threshold incorrectly classifies the pairwise locations A_2 and B_2 as "not together", because these locations were recorded 3-min apart. A dynamic threshold of $d(A_2B_2) < [30\ m + d(A_1A_2) + d(B_1B_2)]$ classifies them correctly as "together".

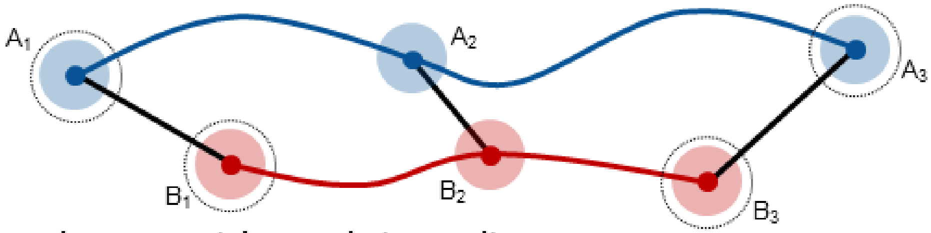

③ **Pair members separately move between sites**

A dynamic threshold misclassifies the pairwise locations A_2 and B_2 as "together", but as none of the pairwise positions were within 30 m the whole bout is classified as "not together".

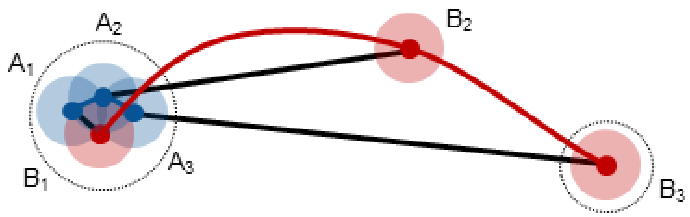

④ **Pair members separate**

A dynamic threshold misclassifies the pairwise locations A_2 and B_2 as "together", but this is corrected given that the birds are not together during the next time interval (A_3, B_3).

Figure S1. Graphical illustration of different scenarios to define whether two birds (e.g. a breeding pair) were "together" or "not together" based on three subsequent GPS locations (points) from a male (A_1 to A_3) and a female (B_1 to B_3) red phalarope. GPS fixes were typically obtained at 10-min intervals. When a pair was caught together, we started their tags at the exact same time, but in general the fixes were not synchronised between the two individuals. In the illustrated example, the female GPS locations were recorded 3 min after the male GPS locations. Thus, we first defined "pair-wise" locations as any two GPS fixes recorded closest in time between the two individuals, excluding any that were more than 10 min apart (here: A_1B_1, A_2B_2 A_3B_3). In our dataset, 50% of pair-wise male-female positions were within 2.6 min and 95% within 5 min. In scenario ①, the birds stayed together in the same site (dotted circle, left) or they stayed separately at two different sites (right) over time. In this scenario, we can then define birds as "together" or "not together" based on a fixed threshold of 30 m (i.e. locations within 30 m are classified as together, d(AB) < 30 m), as illustrated by the overlap in the 15 m light red and light blue area around the points. This threshold was based on data on tag accuracy (Figure S2), as well as direct observations of breeding pairs showing that they usually stay within 15 m of each other when they are together, i.e. they were in visual and acoustical contact (with frequent low amplitude contact calls). However, using a fixed threshold leads to false negative ("not together") classifications when birds move together, as illustrated in scenario ②. In this example, the pair moved together (blue and red lines) from one site (A_1B_1) to a different site (A_3B_3). Using a fixed threshold would classify the pair as "together" at the beginning and end of the flight when they were stationary (because $d(A_1B_1)$ and $d(A_3B_3) < 30$ m), but as "not together" during the flight, because the female's position was recorded 3 min after the male's position (e.g. if the birds would have moved in one direction at a speed of 15 km/h, a 3 min delay in fixes would locate the female 750 m from the male, such that $d(A_2B_2) = 750$ m). Therefore, we used a "dynamic threshold" to define locations as "together" or not. This dynamic threshold includes the fixed threshold of 30 m, but additionally takes the distances that were moved by each individual between the previous and the current position into account. Thus, the birds are classified as "together" when $d(A_2B_2) < [30 \text{ m} + d(A_1A_2) + d(B_1B_2)]$. However, this dynamic threshold is too lenient, because it can lead to false positives (i.e. it misclassifies individuals as together when they are in fact separated), as illustrated in scenario ③. To correct these misclassifications, we used the additional rule that each bout of being "together" (a continuum of pair-wise locations classified as "together", separated by pair-wise locations that are classified as "not together") needs to contain at least one instance where the distance between the two birds was smaller than the fixed threshold (i.e. $d(A_i,B_i) < 30$m). Otherwise, the entire bout is classified as birds being "not together". This approach leaves one last problem unsolved, which is illustrated in scenario ④. When one or both birds fly away from each other after having been together, the dynamic threshold will still classify the birds as

“together” (e.g. A_2B_2) because the bird that left is still within the dynamic threshold distance, even though they are no longer together. To solve this issue, we defined the last pairwise location in a bout of “together” observations as “not together” when it was above the fixed threshold of 30 m. The procedure described here yielded a classification that corresponds to our intuitive judgment (see supplementary video).

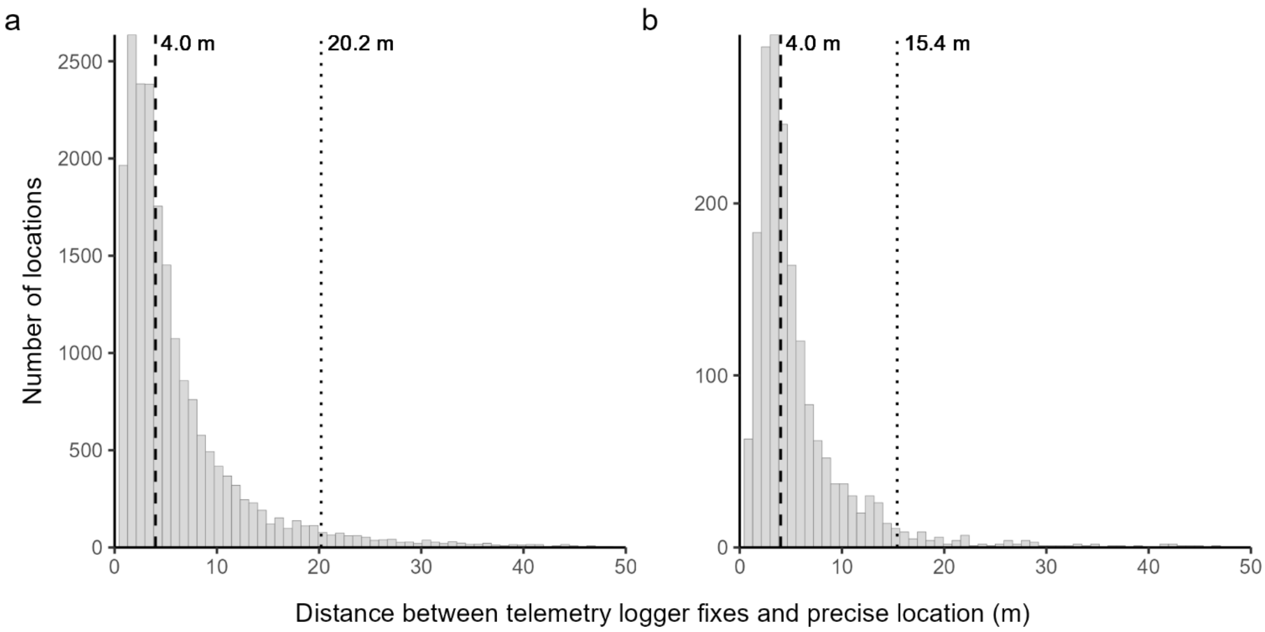

Figure S2. Illustration of the accuracy of the NanoRadioTag-3 (Milsar Inc.) telemetry loggers. Shown are the distances between the loggers' GPS fixes and the precise known locations. (a) Data from 10 loggers that were placed in our study site at a fixed location for 15 days. Of a total of 20150 recorded locations, 95% were within 20.2 m of the precise location, and the median distance was 4.0 m. Overall, 0.7% (N = 133) of the recorded locations were > 50 m away from the precise location (maximum = 398 m) and these were excluded from the plot. (b) Data from one male red phalarope with a logger while incubating (exact location of nest known). We extracted periods of incubation based on data from a temperature logger placed in the nest (using a 30°C threshold). Of a total of 1846 positions recorded during incubation, 95% were within 15.4 m of the precise nest location and the median distance was 4.0 m. Overall, 0.3% (N = 5) of the recorded incubation positions were > 50 m from the nest (max = 90 m) and these were excluded from the plot. At each fixed location or nest, we determined the precise coordinates using waypoint averaging for 10 min with a Garmin Oregon 700.

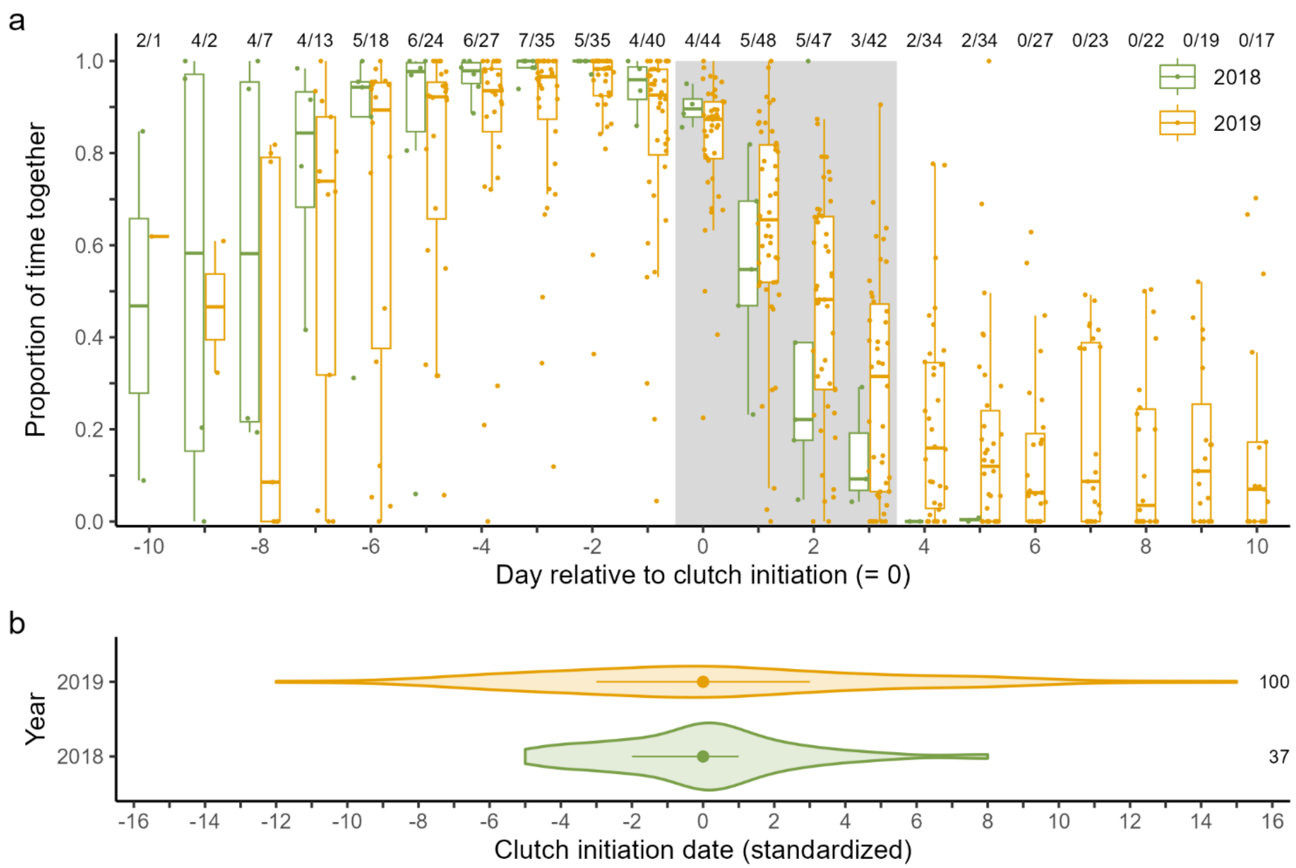

Figure S3. (a) Proportion of time male and female red phalaropes spent together in 2018 and 2019 in relation to the start of egg laying, i.e. day relative to the initiation of the clutch of the focal pair. The data are from a total of 64 breeding pairs (68 clutches). Shown are box plots with the median (centre line), 25–75th percentile (limits), minimum and maximum values without outliers (whiskers), and raw data for each day by breeding pair (dots). The grey shaded area indicates the egg laying period, assuming a typical clutch of four eggs. Numbers on top indicate the number of nests with data for each day and year. (b) Distribution of clutch initiation dates (standardized by subtracting the mean of each year) within the intensive study plot (see methods) for 2018 and 2019. Shown are the median (dot), 25–75th percentile (lines) and a violin plot illustrating kernel probability densities, i.e. the width of the grey area represents the proportion of data located there (maximum width: 11 nests in 2018 and 10 nests in 2019). The difference in the length of the breeding season between the years was due to an exceptionally late snow melt in 2018.

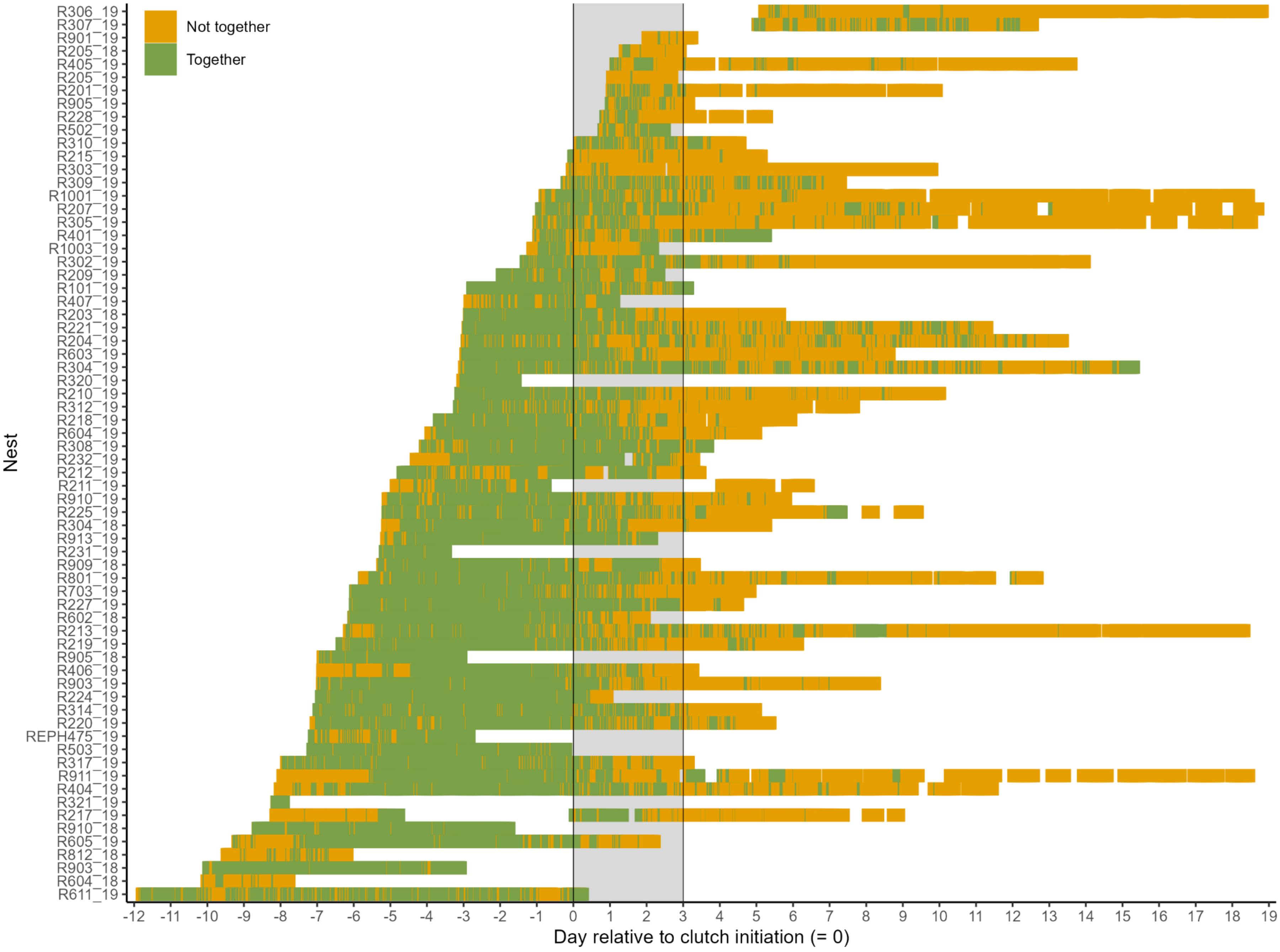
Not together
Together
Nest
R306_19
R307_19
R901_19
R205_18
R405_19
R205_19
R201_19
R905_19
R228_19
R502_19
R310_19
R215_19
R303_19
R309_19
R1001_19
R207_19
R305_19
R401_19
R1003_19
R302_19
R209_19
R101_19
R407_19
R203_18
R221_19
R204_19
R603_19
R304_19
R320_19
R210_19
R312_19
R218_19
R604_19
R308_19
R232_19
R212_19
R211_19
R910_19
R225_19
R304_18
R913_19
R231_19
R909_18
R801_19
R703_19
R227_19
R602_18
R213_19
R219_19
R905_18
R406_19
R903_19
R224_19
R314_19
R220_19
REPH475_19
R503_19
R317_19
R911_19
R404_19
R321_19
R217_19
R910_18
R605_19
R812_18
R903_18
R604_18
R611_19
-12 -11 -10 -9 -8 -7 -6 -5 -4 -3 -2 -1 0 1 2 3 4 5 6 7 8 9 10 11 12 13 14 15 16 17 18 19
Day relative to clutch initiation (= 0)

Figure S4. Overview showing whether the members of a red phalarope breeding pair were together or not for each of 68 nests (from 64 breeding pairs). Data are shown for each 10 min-period in relation to the start of egg laying, i.e. the day relative to the initiation of the clutch of the focal pair. The grey shaded area indicates the egg laying period, assuming a typical clutch of four eggs. Each nest is represented by a bar and white indicates missing data.

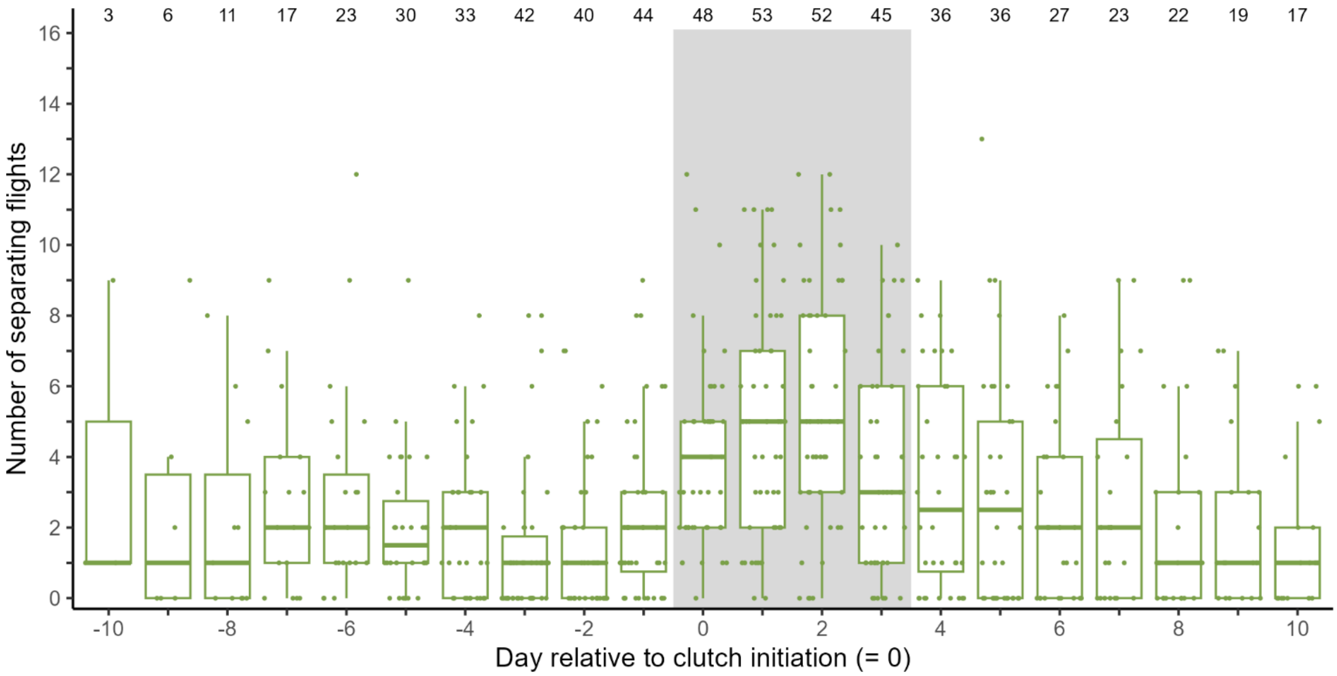

Figure S5. Number of separating flights in which the female or the male moved away from its mate in relation to the start of egg laying, i.e. day relative to the initiation of the clutch of the focal pair. Shown are box plots with the median (centre line), 25–75th percentile (limits), minimum and maximum values without outliers (whiskers), and raw data for each day by breeding pair (dots). The grey shaded area indicates the egg laying period, assuming a typical clutch of four eggs. Numbers on top indicate the number of nests with data for each day.

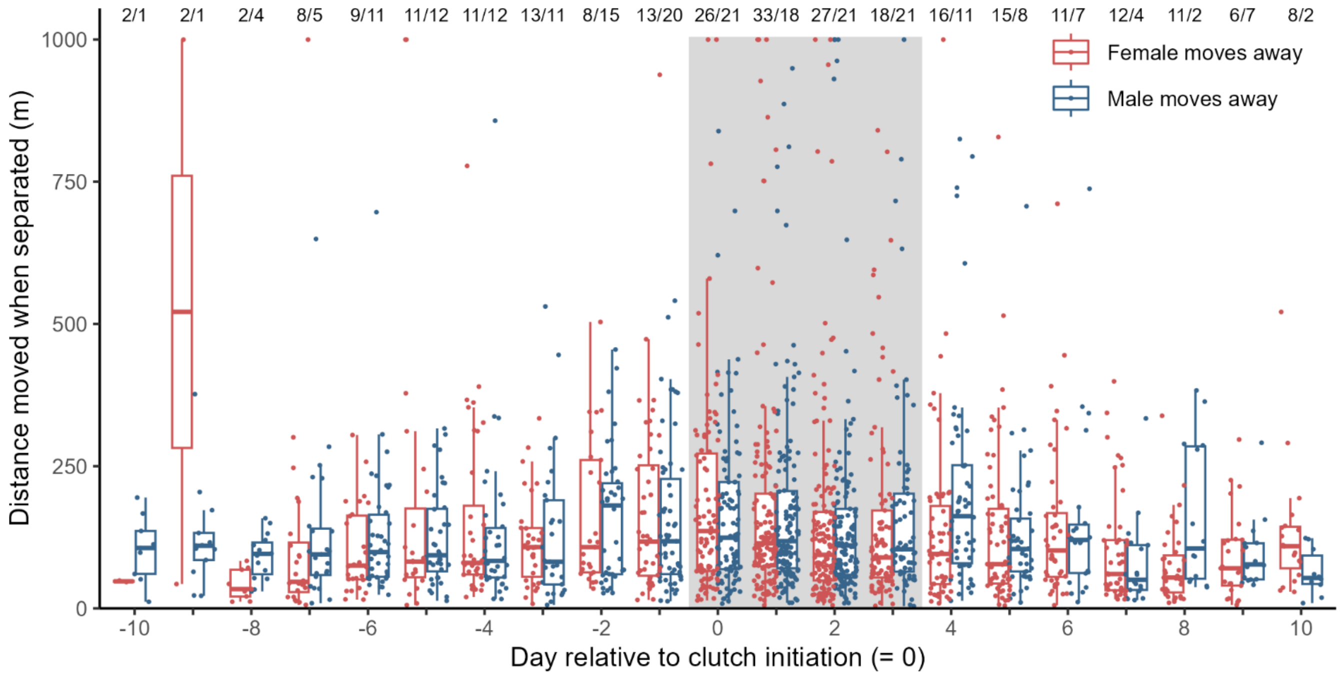

Figure S6. Distances moved by the sex that was responsible for the separation of the breeding pair in relation to the start of egg laying, i.e. day relative to the initiation of the clutch of the focal pair. Box plots show the median (centre line), 25–75th percentile (limits), minimum and maximum values without outliers (whiskers), and raw data for each day and breeding pair (dots). Distances > 1000 m were set to 1000 m.

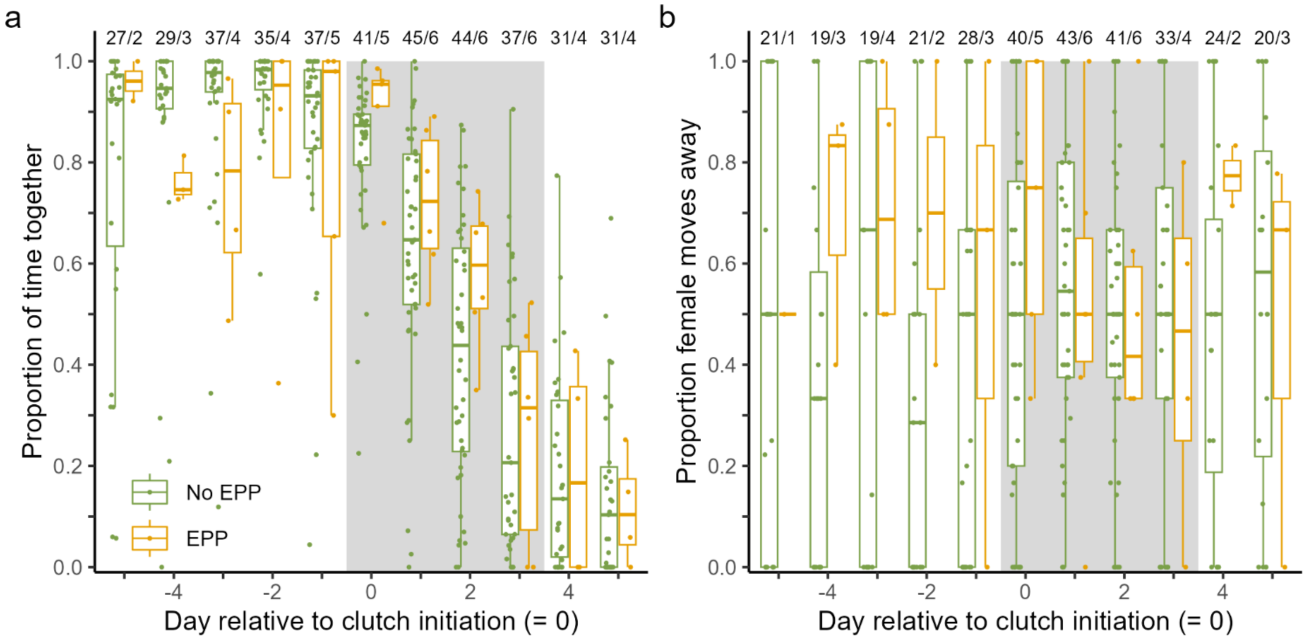

Figure S7. (a) Proportion of time red phalarope pair members spent together in relation to the start of egg laying (i.e. day relative to the initiation of the clutch of the focal pair) for pairs with and without extra-pair paternity (EPP; i.e. whether at least one egg in the focal clutch was sired by an extra-pair male). (b) Proportion of the total number of separating flights in which the female moved away from the male in relation to the start of egg laying (i.e. the day relative to the initiation of the clutch of the focal pair) for pairs with and without extra-pair paternity. Shown are box plots with the median (centre line), 25–75th percentile (limits), minimum and maximum values without outliers (whiskers), and raw data for each day by breeding pair (dots). The grey shaded area indicates the egg laying period, assuming a typical clutch of four eggs. Numbers on top indicate the number of nests with data for each day and category.

Supplementary Tables

Table S1. Results of a generalized linear mixed model with the binary variable “together” (yes/no at a given 10-min interval) as the dependent variable and day relative to clutch initiation (i.e. day on which the first egg was laid = 0), clutch initiation date (standardized by subtracting the mean of each year) and time (as sinus and cosinus) as explanatory variables. Data are from the pre-laying period (days -5 to -1). We included nest ID as a random effect allowing for random slopes over the day relative to clutch initiation. $N_{Nests} = 51$, $N_{males} = 49$, $N_{females} = 48$ and $N_{Observations} = 18289$.

**Parameter**	**Estimate**	**SE**	**Statistic**	***p***
Intercept	2.825	0.255	11.071	<0.001
Sin(time)	0.318	0.045	7.126	<0.001
Cos(time)	0.185	0.042	4.421	<0.001
Clutch initiation date (linear)	55.914	30.779	1.817	0.07
Clutch initiation date (quadratic)	-100.626	28.965	-3.474	0.001
Day relative to clutch initiation (linear)	130.976	46.028	2.846	0.004
Day relative to clutch initiation (quadratic)	-83.673	5.599	-14.946	<0.001
Random intercept	4.278			
R^2 conditional	0.859			
R^2 marginal	0.074			

Table S2. Results of a generalized linear mixed model with the binary variable "together" (yes/no at a given 10-min interval) as the dependent variable and day relative to clutch initiation (i.e. day on which the first egg was laid = 0), clutch initiation date (standardized by subtracting the mean of each year) and time (as sinus and cosinus) as explanatory variables. Data are from the pre-laying period (days 0 to 3). We included nest ID as a random effect allowing for random slopes over the day relative to clutch initiation. N_{Nests} = 56, N_{males} = 53, $N_{females}$ = 50 and $N_{Observations}$ = 19311.

**Parameter**	**Estimate**	**SE**	**Statistic**	***p***
Intercept	0.277	0.146	1.903	0.06
Sin(time)	-0.029	0.026	-1.114	0.27
Cos(time)	-0.215	0.025	-8.591	<0.001
Clutch initiation date (linear)	17.239	18.946	0.910	0.36
Clutch initiation date (quadratic)	-74.809	17.163	-4.359	<0.001
Day relative to clutch initiation (linear)	-175.554	16.447	-10.674	<0.001
Day relative to clutch initiation (quadratic)	-5.892	3.043	-1.936	0.05
Random intercept	1.057			
R^2 conditional	0.480			
R^2 marginal	0.303			

Table S3. Results of a generalized linear mixed model with the proportion of time spent together per day as the dependent variable and day relative to clutch initiation (i.e. day on which the first egg was laid = 0), clutch initiation date (standardized by subtracting the mean of each year) and year (2018 and 2019) as explanatory variables. Data are from the pre-laying period (days -5 to -1). We included nest ID as a random effect allowing for random slopes over the day relative to clutch initiation. N_{Nests} = 51 (2018: 7, 2019: 44), N_{males} = 49, $N_{females}$ = 48 and $N_{Observations}$ = 189 (2018: 28, 2019:161).

Parameter	Estimate	SE	Statistic	*p*
Intercept	2.390	0.456	5.247	<0.001
Day relative to clutch initiation (linear)	5.147	2.512	2.049	0.04
Day relative to clutch initiation (quadratic)	-4.947	1.222	-4.049	<0.001
Clutch initiation date (linear)	1.499	1.590	0.943	0.35
Clutch initiation date (quadratic)	-6.715	1.723	-3.897	<0.001
Year (2019)	-0.418	0.402	-1.039	0.30
Random intercept	1.701			
R^2 conditional	1.050			
R^2 marginal	0.156			

Table S4. Results of a generalized linear mixed model with the proportion of time spent together per day as the dependent variable and day relative to clutch initiation (i.e. day on which the first egg was laid = 0), clutch initiation date (standardized by subtracting the mean of each year) and year (2018 and 2019) as explanatory variables. Data are from the egg-laying period (days 0 to 3). We included nest ID as a random effect allowing for random slopes over the day relative to clutch initiation. N_{Nests} = 56 (2018: 5, 2019: 51), N_{males} = 53, $N_{females}$ = 50 and $N_{Observations}$ = (2018: 17, 2019: 181).

**Parameter**	**Estimate**	**SE**	**Statistic**	***p***
Intercept	1.315	0.432	3.040	0.002
Day relative to clutch initiation	-0.877	0.079	-11.063	<0.001
Clutch initiation date (linear)	5.372	1.918	2.801	0.005
Clutch initiation date (quadratic)	-5.714	1.651	-3.462	0.001
Year (2019)	0.097	0.446	0.218	0.83
Random intercept	0.548			
R^2 conditional	1.035			
R^2 marginal	0.570			

Table S5. Results of a generalized linear mixed model with the proportion of time spent together per day as the dependent variable and day relative to clutch initiation (i.e. day on which the first egg was laid = 0) and clutch initiation date (standardized by subtracting the mean of each year) as explanatory variables. Data are from the pre-laying period (days -5 to -1). We included nest ID as a random effect allowing for random slopes over the day relative to clutch initiation. N_{Nests} = 51, N_{males} = 49, $N_{females}$ = 48 and $N_{Observations}$ = 189.

**Parameter**	**Estimate**	**SE**	**Statistic**	***p***
Intercept	1.997	0.164	12.197	<0.001
Day relative to clutch initiation (linear)	4.764	2.145	2.221	0.03
Day relative to clutch initiation (quadratic)	-4.925	1.117	-4.408	<0.001
Clutch initiation date (linear)	1.536	1.450	1.059	0.30
Clutch initiation date (quadratic)	-6.973	1.570	-4.441	<0.001
Random intercept	1.599			
R^2 conditional	1.063			
R^2 marginal	0.161			

Table S6. Results of a generalized linear mixed model with the proportion of time spent together per day as the dependent variable and day relative to clutch initiation (i.e. day on which the first egg was laid = 0) and clutch initiation date (standardized by subtracting the mean of each year) as explanatory variables. Data are from the egg-laying period (days 0 to 3). We included nest ID as a random effect allowing for random slopes over the day relative to clutch initiation. $N_{Nests} = 56$, $N_{males} = 53$, $N_{females} = 50$ and $N_{Observations} = 198$.

**Parameter**	**Estimate**	**SE**	**Statistic**	***p***
Intercept	1.405	0.130	10.784	<0.001
Day relative to clutch initiation	-0.878	0.079	-11.100	<0.001
Clutch initiation date (linear)	5.348	1.906	2.806	0.005
Clutch initiation date (quadratic)	-5.632	1.612	-3.494	<0.001
Random intercept	0.536			
R^2 conditional	1.035			
R^2 marginal	0.574			

Table S7. Results of a generalized linear mixed model with the proportion of time spent together per day as the dependent variable and day relative to clutch initiation (i.e. day on which the first egg was laid = 0), clutch initiation date (standardized by subtracting the mean of each year) and data type (breeding pair / random pair) in interaction with both as explanatory variables. Data are from the pre-laying period (days -5 to -1). We included nest ID as a random effect allowing for random slopes over the day relative to clutch initiation. N_{Nests} = 51 (+178 random pair comparisons), N_{males} = 57, $N_{females}$ = 54 and $N_{Observations}$ = 189 (+239 random pair comparisons).

**Parameter**	**Estimate**	**SE**	**Statistic**	***p***
Intercept	2.029	0.115	17.664	<0.001
Day relative to clutch initiation (linear)	7.692	2.485	3.095	0.002
Day relative to clutch initiation (quadratic)	-7.819	1.491	-5.243	<0.001
Data type (random pairs)	-4.126	0.150	-27.570	<0.001
Clutch initiation date (linear)	9.207	2.113	4.357	<0.001
Clutch initiation date (quadratic)	-14.289	2.070	-6.901	<0.001
Clutch initiation date (linear):data type (random pairs)	-7.978	2.554	-3.124	0.002
Clutch initiation date (quadratic):data type (random pairs)	13.221	2.428	5.444	<0.001
Random intercept	1.029			
R^2 conditional	1.003			
R^2 marginal	0.832			

Table S8. Results of a generalized linear mixed model with the proportion of time spent together per day as the dependent variable and day relative to clutch initiation (i.e. day on which the first egg was laid = 0), clutch initiation date (standardized by subtracting the mean of each year) and data type (breeding pair / random pair) in interaction with both as explanatory variables. Data are from the egg-laying period (days 0 to 3). We included nest ID as a random effect allowing for random slopes over the day relative to clutch initiation. N_{Nests} = 56 (+145 random pair comparisons), N_{males} = 58, $N_{females}$ = 54 and $N_{Observations}$ = 198 (+186 random pair comparisons).

**Parameter**	**Estimate**	**SE**	**Statistic**	***p***
Intercept	1.352	0.123	11.029	<0.001
Day relative to clutch initiation	-0.859	0.062	-13.910	<0.001
Data type (random pairs)	-3.391	0.172	-19.686	<0.001
Clutch initiation date (linear)	5.189	2.116	2.452	0.014
Clutch initiation date (quadratic)	-7.044	1.950	-3.612	<0.001
Day relative to clutch initiation:data type (random pairs)	0.812	0.088	9.250	<0.001
Clutch initiation date (linear):data type (random pairs)	0.190	2.604	0.073	0.94
Clutch initiation date (quadratic):data type (random pairs)	6.304	2.644	2.384	0.02
Random intercept	0.482			
R^2 conditional	0.949			
R^2 marginal	0.728			

Table S9. Results of a generalized linear mixed model with the proportion of time spent together per day as the dependent variable and day relative to clutch initiation (i.e. day on which the first egg was laid = 0), clutch initiation date (standardized by subtracting the mean of each year) and data type (breeding pair / random pair) in interaction with both as explanatory variables. Data are from after the egg-laying period (days 4 to 10). We included nest ID as a random effect allowing for random slopes over the day relative to clutch initiation. N_{Nests} = 38 (+174 random pair comparisons), N_{males} = 48, $N_{females}$ = 46 and $N_{Observations}$ = 180 (+309 random pair comparisons).

**Parameter**	**Estimate**	**SE**	**Statistic**	***p***
Intercept	-1.297	0.294	-4.412	<0.001
Day relative to clutch initiation	-0.148	0.044	-3.334	0.001
Data type (random pairs)	-0.569	0.376	-1.512	0.13
Clutch initiation date	-0.155	0.031	-4.973	<0.001
Day relative to clutch initiation:data type (random pairs)	0.121	0.055	2.206	0.03
Clutch initiation date:data type (random pairs)	0.158	0.030	5.207	<0.001
Random intercept	0.744			
R^2 conditional	0.351			
R^2 marginal	0.097			

Table S10. Results of a generalized linear mixed model with the binary variable “female move” (yes/no) as the dependent variable and day relative to clutch initiation (i.e. day on which the first egg was laid = 0) and clutch initiation date (standardized by subtracting the mean of each year) as explanatory variables. Data are from the pre-laying period (-5 to -1). We included nest ID as a random effect allowing for random slopes over the day relative to clutch initiation. N_{Nests} = 46, N_{males} = 46, $N_{females}$ = 44 and $N_{Observations}$ = 355.

**Parameter**	**Estimate**	**SE**	**Statistic**	***p***
Intercept	-0.304	0.287	-1.059	0.29
Day relative to clutch initiation	-0.087	0.085	-1.025	0.31
Clutch initiation date	-0.124	0.048	-2.584	0.01
Random intercept	0.754			
R^2 conditional	0.144			
R^2 marginal	0.055			

Table S11. Results of a generalized linear mixed model with the binary variable “female move” (yes/no) as the dependent variable and day relative to clutch initiation (i.e. day on which the first egg was laid = 0) and clutch initiation date (standardized by subtracting the mean of each year) as explanatory variables. Data are from the egg-laying period (days 0 to 3). We included nest ID as a random effect allowing for random slopes over the day relative to clutch initiation. N_{Nests} = 56, N_{males} = 53, $N_{females}$ = 50 and $N_{Observations}$ = 906.

**Parameter**	**Estimate**	**SE**	**Statistic**	***p***
Intercept	-0.053	0.164	-0.321	0.75
Day relative to clutch initiation	0.086	0.079	1.097	0.27
Clutch initiation date	0.045	0.028	1.643	0.10
Random intercept	0.710			
R^2 conditional	0.083			
R^2 marginal	0.008			

Table S12. Results of a generalized linear mixed model with the distance moved away when separated (in m) as the dependent variable and with sex, relative clutch initiation day (i.e. day on which the first egg was laid = 0) and day relative to clutch initiation (standardized by subtracting the mean of each year) as explanatory variables. Data are from pre-laying period (days -5 to -1). We included nest ID as a random effect allowing for random slopes over the day relative to clutch initiation. N_{Nests} = 46, N_{males} = 46, $N_{females}$ = 44 and $N_{Observations}$ = 355.

**Parameter**	**Estimate**	**SE**	**Statistic**	***p***
Intercept	176.404	20.272	8.702	<0.001
Sex (male)	-29.511	16.525	-1.786	0.07
Day relative to clutch initiation	4.284	6.687	0.641	0.52
Clutch initiation date	4.556	3.440	1.325	0.19
Random intercept	14.205			
R^2 conditional	0.197			
R^2 marginal	0.015			

Table S13. Results of a generalized linear mixed model with the distance moved away when separated (in m) as the dependent variable and with sex, relative clutch initiation day (i.e. day on which the first egg was laid = 0) and day relative to clutch initiation (standardized by subtracting the mean of each year) as explanatory variables. Data are from egg-laying period (days 0 to 3). We included nest ID as a random effect allowing for random slopes over the day relative to clutch initiation. N_{Nests} = 56, N_{males} = 53, $N_{females}$ = 50 and $N_{Observations}$ = 906.

**Parameter**	**Estimate**	**SE**	**Statistic**	***p***
Intercept	222.160	28.174	7.885	<0.001
Sex (male)	-21.889	15.438	-1.418	0.16
Day relative to clutch initiation	-4.178	7.743	-0.540	0.59
Clutch initiation date	-2.538	6.249	-0.406	0.69
Random intercept	169.618			
R^2 conditional				
R^2 marginal	0.004			

Table S14. Results of a generalized linear mixed model with the proportion of time spent at the nest per dayas the dependent variable and relative clutch initiation day (i.e. day on which the first egg was laid = 0) and day relative to clutch initiation (standardized by subtracting the mean of each year) as explanatory variables. Data are from the egg-laying period (days 0 to 3). We included nest ID as a random effect allowing for random slopes over the day relative to clutch initiation. N_{Nests} = 56, N_{males} = 53, $N_{females}$ = 50 and $N_{Observations}$ = 198.

**Parameter**	**Estimate**	**SE**	**Statistic**	***p***
Intercept	-1.314	0.097	-13.562	<0.001
Day relative to clutch initiation	0.553	0.071	7.753	<0.001
Clutch initiation date	0.039	0.023	1.723	0.09
Random intercept	0.461			
R^2 conditional	0.915			
R^2 marginal	0.301			

Table S15. Results of a generalized linear mixed model with the proportion of time spent at the nest per day as the dependent variable and relative clutch initiation day (i.e. day on which the first egg was laid = 0) and day relative to clutch initiation (standardized by subtracting the mean of each year) as explanatory variables. Data are from the egg-laying period (days 0 to 3). We included nest ID as a random effect allowing for random slopes over the day relative to clutch initiation. N_{Nests} = 56, N_{males} = 53, $N_{females}$ = 50 and $N_{Observations}$ = 198.

**Parameter**	**Estimate**	**SE**	**Statistic**	***p***
Intercept	-1.594	0.101	-15.761	<0.001
Day relative to clutch initiation	-0.251	0.068	-3.671	<0.001
Clutch initiation date (linear)	4.685	1.439	3.255	0.001
Clutch initiation date (quadratic)	-3.947	1.316	-2.999	0.003
Random intercept	0.436			
R^2 conditional	0.785			
R^2 marginal	0.227			

Table S16. Results of a generalized linear mixed model with the proportion of time spent together per day as the dependent variable and relative clutch initiation day (i.e. day on which the first egg was laid = 0), day relative to clutch initiation (standardized by subtracting the mean of each year) and extra-pair paternity (whether at least one egg in the clutch was sired by an extra-pair male, yes/no; EPP) as explanatory variables. Data are from the pre-laying period (days -5 to -1). We included nest ID as a random effect allowing for random slopes over the day relative to clutch initiation. N_{Nests} = 49 (with EPP: 5, without EPP: 44), N_{males} = 47, $N_{females}$ = 46 and $N_{Observations}$ = 183 (with EPP: 18, without EPP: 165).

**Parameter**	**Estimate**	**SE**	**Statistic**	***p***
Intercept	2.019	0.179	11.280	<0.001
Day relative to clutch initiation (linear)	4.847	2.303	2.104	0.04
Day relative to clutch initiation (quadratic)	-4.830	1.143	-4.224	<0.001
Clutch initiation date (linear)	1.463	1.641	0.892	0.37
Clutch initiation date (quadratic)	-7.099	1.737	-4.088	<0.001
EPP (yes)	-0.208	0.409	-0.508	0.61
Random intercept	1.704			
R^2 conditional	1.055			
R^2 marginal	0.143			

Table S17. Results of a generalized linear mixed model with the proportion of time spent together per day as the dependent variable and relative clutch initiation day (i.e. day on which the first egg was laid = 0), day relative to clutch initiation (standardized by subtracting the mean of each year) and extra-pair paternity (whether at least one egg in the clutch was sired by an extra-pair male, yes/no; EPP) as explanatory variables. Data are from the fertile egg-laying period (0 to 2). We included nest ID as a random effect allowing for random slopes over the day relative to clutch initiation. N_{Nests} = 54 (with EPP: 6, without EPP: 48), N_{males} = 51, $N_{females}$ = 48 and $N_{Observations}$ = 147 (with EPP: 17, without EPP: 130).

**Parameter**	**Estimate**	**SE**	**Statistic**	***p***
Intercept	1.425	0.140	10.150	<0.001
Day relative to clutch initiation	-0.935	0.090	-10.414	<0.001
Clutch initiation date (linear)	4.661	1.650	2.824	0.005
Clutch initiation date (quadratic)	-5.518	1.528	-3.612	<0.001
EPP (yes)	0.505	0.413	1.225	0.22
Random intercept	0.619			
R^2 conditional	1.035			
R^2 marginal	0.530			

Table S18. Results of a generalized linear mixed model with the binary variable "female move" (yes/no at a given 10-min interval) as the dependent variable and relative clutch initiation day (i.e. day on which the first egg was laid = 0), day relative to clutch initiation (standardized by subtracting the mean of each year) and extra-pair paternity (whether at least one egg in the clutch was sired by an extra-pair male, yes/no; EPP) as explanatory variables. Data are from the pre-laying period (-5 to -1). We included nest ID as a random effect allowing for random slopes over the day relative to clutch initiation. N_{Nests} = 44 (with EPP: 5, without EPP: 39), N_{males} = 44, $N_{females}$ = 42 and $N_{Observations}$ = 346 (with EPP: 56, without EPP: 290).

**Parameter**	**Estimate**	**SE**	**Statistic**	***p***
Intercept	-0.309	0.302	-1.021	0.31
Day relative to clutch initiation	-0.056	0.088	-0.633	0.53
Clutch initiation date	-0.120	0.051	-2.367	0.02
EPP (yes)	0.692	0.455	1.521	0.13
Random intercept	0.804			
R^2 conditional	0.167			
R^2 marginal	0.088			

Table S19. Results of a generalized linear mixed model with the binary variable "female move" (yes/no at a given 10-min interval) as the dependent variable and relative clutch initiation day (i.e. day on which the first egg was laid = 0), day relative to clutch initiation (standardized by subtracting the mean of each year) and extra-pair paternity (whether at least one egg in the clutch was sired by an extra-pair male, yes/no; EPP) as explanatory variables. Data are from the fertile egg-laying period (0 to 2). We included nest ID as a random effect allowing for random slopes over the day relative to clutch initiation. N_{Nests} = 53 (with EPP: 6, without EPP: 47), N_{males} = 50, $N_{females}$ = 48 and $N_{Observations}$ = 715 (with EPP: 91, without EPP: 624).

**Parameter**	**Estimate**	**SE**	**Statistic**	***p***
Intercept	0.070	0.184	0.381	0.70
Day relative to clutch initiation	0.002	0.112	0.020	0.98
Clutch initiation date	0.023	0.030	0.766	0.44
EPP (yes)	0.113	0.313	0.360	0.72
Random intercept	0.751			
R^2 conditional	0.080			
R^2 marginal	0.002			
