# Supplementary material for: Mutual mate guarding with limited sexual conflict in a sex-role-reversed shorebird
Source: Behav Ecol. 2023 Dec 1;35(1):arad084. doi: 10.1093/beheco/arad084 (PMC10773304; doi:10.1093/beheco/arad084)
Supplement: arad084_suppl_Supplementary_Video_Caption [file arad084_suppl_supplementary_video_caption.pdf]

Electronic supplementary video. Visualization of movements before and during clutch initiation of three red phalarope pairs (0:00 first pair, 2:48 second pair, 5:14 third pair). Females are indicated in red, males in blue and the nest location in brown (turning dark with clutch initiation). The moving bar on top indicates 12 hours before and 12 hours after the present (black bar in the middle); birds classified as together are shown in green and those classified as not together in white. Periods with missing pair-wise data are indicated in grey. On the map, a green ring around the present location (dots) indicates when pair members were classified as together. Eggs appear at the top left around the time when they were approximately laid. Note the running date and time and distance between the pair at the bottom right, and the scale bar at the bottom left. Beige indicates land (i.e. tundra habitat), light blue lakes, darker blue the sea, and grey buildings and roads. Map data are from OpenStreetMap in polar Lambert azimuthal equal area projection with longitude origin 156.65° W (Utqiaġvik).
